# Functionally conserved inner mitochondrial membrane proteins CCDC51 and Mdm33 demarcate a subset of fission events

**DOI:** 10.1083/jcb.202403140

**Published:** 2024-12-24

**Authors:** Alia R. Edington, Olivia M. Connor, Abigail C. Love, Madeleine Marlar-Pavey, Jonathan R. Friedman

**Affiliations:** 1 Department of Cell Biology, University of Texas Southwestern Medical Center, Dallas, TX, USA

## Abstract

While extensive work has examined the mechanisms of mitochondrial fission, it remains unclear whether internal mitochondrial proteins in metazoans play a direct role in the process. Previously, the yeast inner membrane protein Mdm33 was shown to be required for normal mitochondrial morphology and has been hypothesized to be involved in mitochondrial fission. However, it is unknown whether Mdm33 plays a direct role, and it is not thought to have a mammalian homolog. Here, we use a bioinformatic approach to identify a structural ortholog of Mdm33 in humans, CCDC51 (also called MITOK), whose depletion phenocopies loss of Mdm33. We find that knockdown of CCDC51 also leads to reduced rates of mitochondrial fission. Further, we spatially and temporally resolve Mdm33 and CCDC51 to a subset of mitochondrial fission events. Finally, we show that CCDC51 overexpression promotes its spatial association with Drp1 and induces mitochondrial fragmentation, suggesting it is a positive effector of mitochondrial fission. Together, our data reveal that Mdm33 and CCDC51 are functionally conserved and suggest that internal mitochondrial proteins are directly involved in at least a subset of mitochondrial fission events in human cells.

## Teaser

Edington et al. determine that Mdm33 and CCDC51 are functionally conserved mitochondrial inner membrane proteins that are required for normal mitochondrial morphology. The authors show that the proteins spatially demarcate and are required for a subset of mitochondrial division events, suggesting that internal mitochondrial proteins facilitate fission in human cells.

## Introduction

Mitochondria are multifunctional, double membrane–bound organelles whose shape and internal architecture respond to cellular metabolic cues, nutrient availability, and respiratory demands (Bennett et al., 2022; Wai and Langer, 2016). The overall distribution of the organelle arises from dynamic events, including cytoskeletal-based motility, fusion, and fission (Quintana-Cabrera and Scorrano, 2023; Ul Fatima and Ananthanarayanan, 2023). Additionally, the inner mitochondrial membrane (IMM) is dynamic and invaginates to form cristae that house the membrane-shaping and ATP-producing oxidative phosphorylation machinery (Klecker and Westermann, 2021). Despite our extensive knowledge of the mechanisms underlying mitochondrial dynamics and the identification of determinants of internal mitochondrial organization, we still have a poor understanding of how each is coordinated to maintain organelle homeostasis.

In the case of mitochondrial fission, a conserved dynamin superfamily member (Drp1 in humans, Dnm1 in yeast) is recruited to the outer mitochondrial membrane (OMM), oligomerizes to encircle the organelle, and utilizes GTP hydrolysis to constrict and divide the membranes (Kraus et al., 2021). There is precise spatial control of mitochondrial fission, and most sites are pre-marked at the OMM by inter-organelle contacts with the ER (Friedman et al., 2011; Kleele et al., 2021). Mitochondrial fission is also spatially coordinated with mitochondrial DNA (mtDNA) replication events that occur in the matrix (Lewis et al., 2016). Thus, fission must be coordinated across both the OMM and IMM, which raises the question of whether internal mitochondrial proteins are involved in or required for mitochondrial fission.

Recently, we and others identified the first internal mitochondrial protein that is required for mitochondrial fission in fungal species, the intermembrane space (IMS)-localized protein Mdi1 (also called Atg44) (Connor et al., 2023; Fukuda et al., 2023). Without Mdi1, Dnm1 can constrict the organelle but fails to complete fission of the OMM or IMM (Connor et al., 2023; Furukawa et al., 2024). As Mdi1 is not found in metazoan species, it remains an open question whether a functionally equivalent protein is required for division in higher eukaryotes. However, as Dnm1 requires Mdi1 in yeast, it is plausible that human Drp1 may not be able to sever both the IMM and OMM without the coordinated activity of internal factors.

Another internal mitochondrial protein that was previously implicated in mitochondrial fission is the yeast protein Mdm33 (also called She9) (Klecker et al., 2015; Messerschmitt et al., 2003). Mdm33 is an integral IMM protein that is required for normal mitochondrial morphology, and ∆*mdm33* yeast cells form elongated, hollow mitochondria that are partially resistant to the induction of fission (Klecker et al., 2015; Messerschmitt et al., 2003). Additionally, Mdm33 has extensive coiled-coil domains on both the matrix and IMS sides of the IMM and self-associates to form high molecular weight assemblies and is thus hypothesized to coordinate fission across the IMM (Messerschmitt et al., 2003). Furthermore, MDM33 genetically interacts with genes involved in phospholipid synthesis (Hoppins et al., 2011; Klecker et al., 2015), suggesting that Mdm33 may coordinate lipid homeostasis pathways to influence mitochondrial architecture and/or fission dynamics (Klecker et al., 2015). However, Mdm33 is not thought to be conserved outside of fungal species, and its precise role remains elusive despite the unique phenotypes associated with its loss.

Here, we identify a structurally similar human IMM protein to Mdm33, CCDC51. We find that loss of CCDC51 phenocopies mitochondrial morphology defects of ∆*mdm33* yeast cells and that exogenous CCDC51 can partially rescue the loss of Mdm33, demonstrating the proteins are functional orthologs. We observe that acute CCDC51 depletion leads to mitochondrial hyperfusion and reduced mitochondrial fission rates. We also find that Mdm33 in yeast and CCDC51 in human cells are spatially and temporally linked to a subset of mitochondrial fission events. Finally, we show that overexpression of CCDC51 promotes its spatial association with Drp1 and leads to mitochondrial fragmentation. Thus, we have identified a human IMM protein that demarcates, and is a positive effector of, Drp1-mediated mitochondrial fission.

## Results and discussion

### Mdm33 and CCDC51 are conserved mediators of mitochondrial morphology

**Figure 1.**
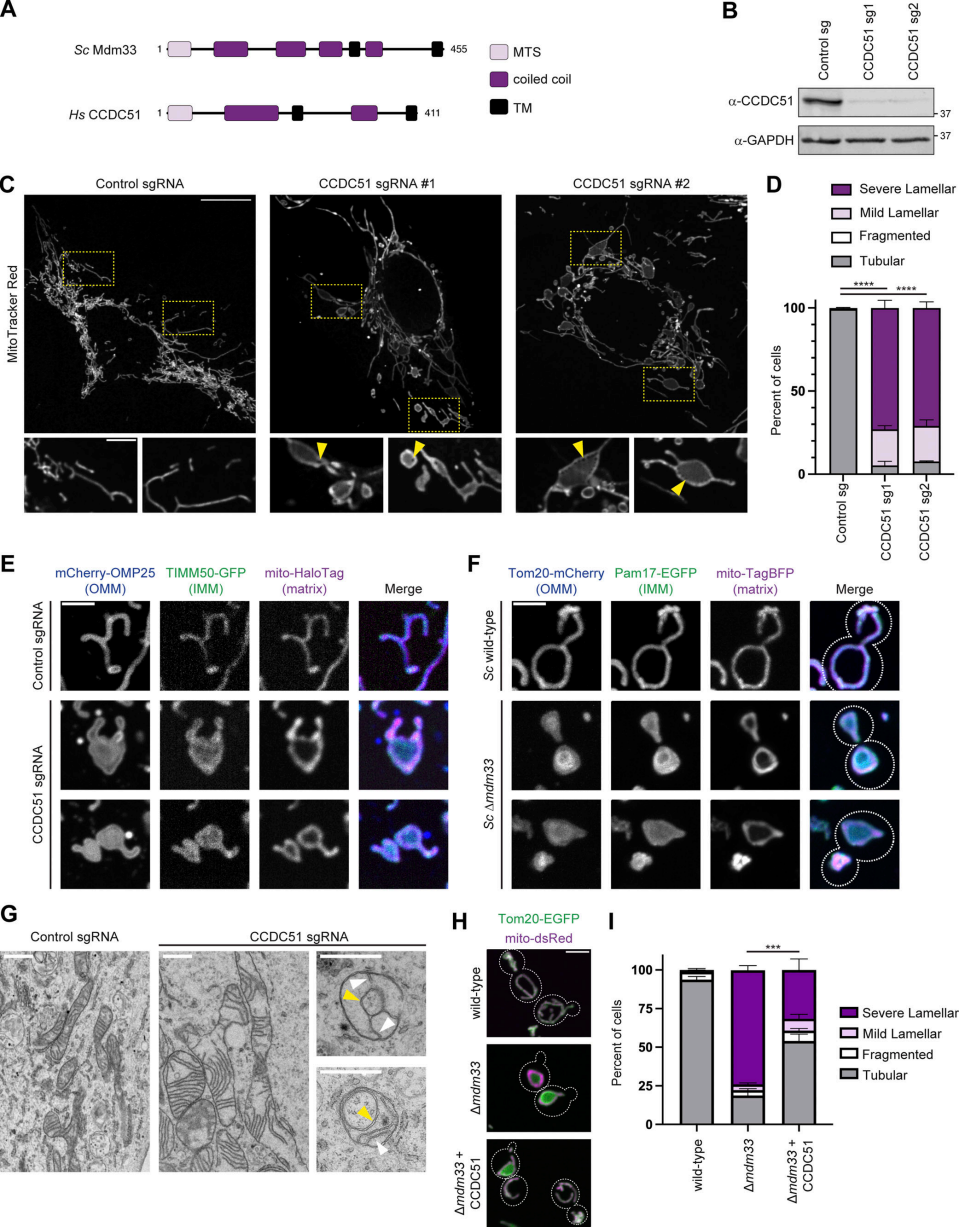
**Mdm33 and CCDC51 are conserved mediators of mitochondrial morphology. (A)** A schematic depicting the predicted domain architectures of yeast Mdm33 and human CCDC51. **(B)** Western analysis with the indicated antibodies of whole-cell lysates from U2OS CRISPRi cells expressing control sgRNA or sgRNAs targeting CCDC51. **(C)** Representative maximum intensity projection confocal images of U2OS CRISPRi cells expressing control or CCDC51-targeted sgRNAs and stained with MitoTracker Red. Insets below are single-plane images and correspond to the indicated dashed boxes. Arrows mark discontinuities in MitoTracker staining. **(D)** A graph of mitochondrial morphology categorization from the indicated CRISPRi cells as in C. Data shown represent 100 cells per condition in each of three independent experiments, and bars indicate SEM. Asterisks (****P < 0.0001) represent unpaired two-tailed *t* tests of tubular mitochondrial morphology. **(E)** Representative maximum intensity projection confocal images are shown of the indicated CRISPRi cells expressing mCherry-OMP25, TIMM50-GFP, and mito-HaloTag labeled with JF646. **(F)** Maximum intensity projection confocal images are shown of the indicated yeast strains expressing Tom20-mCherry, Pam17-EGFP, and mito-TagBFP. Dashed lines indicate cell outlines. **(G)** EM images are shown of mitochondria from U2OS CRISPRi cells expressing control or CCDC51 sgRNA, where indicated. Yellow arrows mark internal ring structures and white arrows mark cristae. **(H)** Maximum intensity projection confocal images are shown of the indicated yeast strains-expressing Tom20-EGFP and mito-dsRed. Human CCDC51 is expressed under the control of an estradiol-driven promoter, and all strains were grown constitutively in the presence of 20 nM estradiol. Dashed lines indicate cell outlines. **(I)** A graph of the categorization of mitochondrial morphology from cells as in H. Data shown represent 75 cells per condition in each of three independent experiments, and bars indicate SEM. Asterisks (***P < 0.001) represent an unpaired two-tailed *t* test of tubular mitochondrial morphology. Scale bars = (C) 15 µm (5 µm in insets); (E and F) 3 µm; (G) 800 nm; and (H) 3 µm. Source data are available for this figure: SourceData F1.

**Figure S1.**
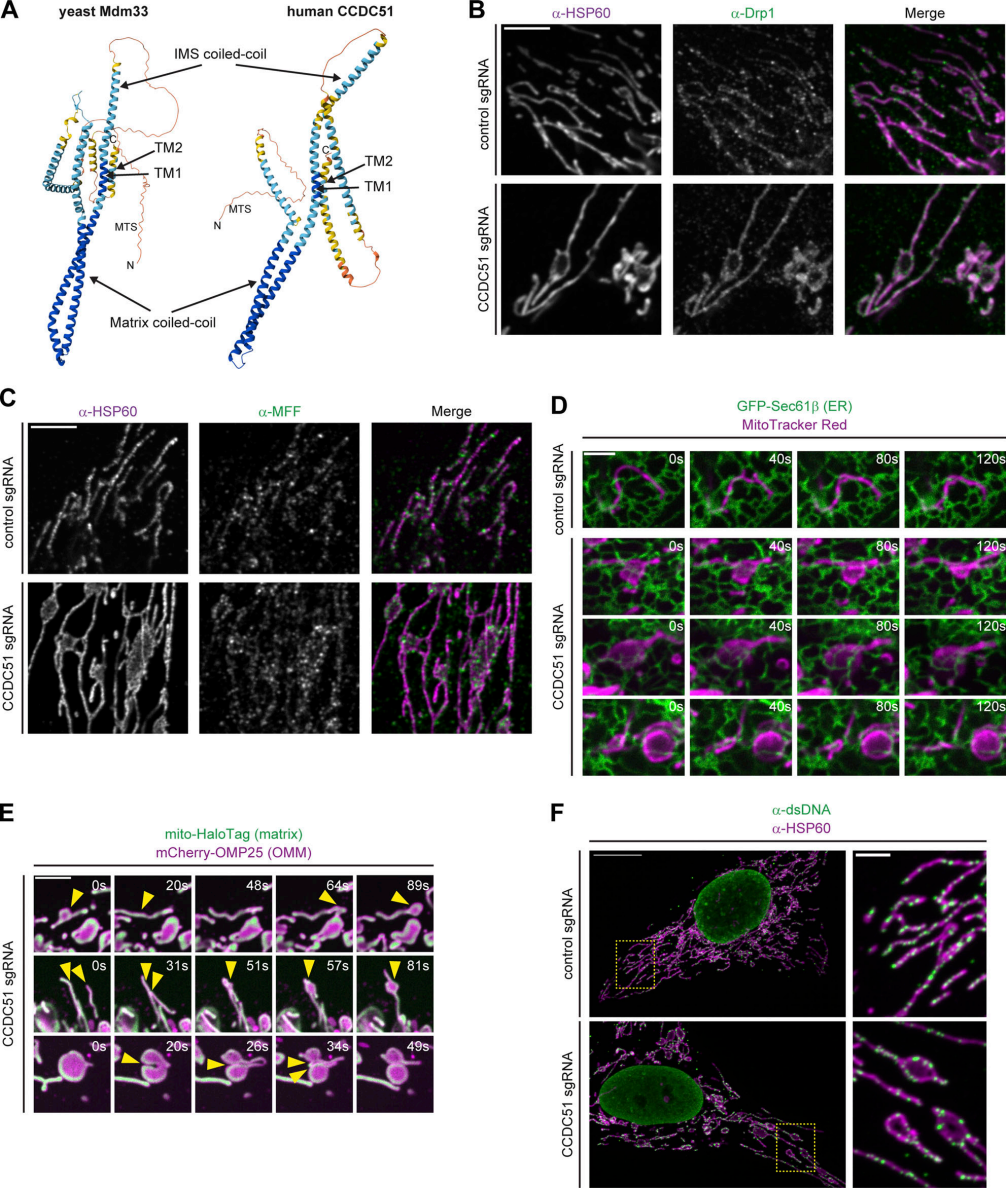
**Additional characterization of CCDC51 CRISPRi cells. (A)** AlphaFold2-generated predicted structures with annotations of predicted MTS, TM domains (TM1 and TM2), and coiled-coil domains shown for yeast Mdm33 (left) and human CCDC51 (right). Note that the orientation of TM2 is likely reversed. **(B and C)** Representative single-plane confocal images are shown of control (top) and CCDC51 CRISPRi cells (bottom) that were fixed and immunolabeled for HSP60 and (B) Drp1 or (C) MFF. **(D)** Single-plane confocal images are shown at the indicated time intervals of control (top) or CCDC51 CRISPRi (bottom) cells expressing GFP-Sec61β and stained with MitoTracker Red. **(E)** Single-plane confocal time-lapse images at the indicated intervals are shown of CCDC51 CRISPRi cells expressing mCherry-OMP25 and mito-HaloTag/JF646. Arrows mark sites of resolution and reformation of lamellar mitochondria (top row), an example of formation of a lamellar mitochondrion via apparent fusion (middle row), and an example of fission of a lamellar mitochondrion (bottom row). See also Videos 1, 2, and 3. **(F)** Representative maximum intensity projection confocal images are shown of control (top) and CCDC51 (bottom) CRISPRi cells fixed and immunolabeled for HSP60 and dsDNA. Insets at the right correspond to the indicated dashed boxes. Scale bars: (B–D) 5 µm; (E) 3 µm; and (F) 15 µm (3 µm in inset).

Mdm33 consists of an N-terminal mitochondrial targeting sequence (MTS) and two transmembrane (TM) domain segments that are interspersed with predicted coiled-coil domains (Messerschmitt et al., 2003) (Fig. 1 A). Given that these Mdm33-defining domains are elements that may be under less evolutionary pressure to maintain primary sequence homology or may play a structural role (Surkont and Pereira-Leal, 2015; Truebestein and Leonard, 2016), we reasoned that a potential functional ortholog may exist with minimal sequence similarity. As expected, a BLAST search failed to identify any metazoan sequence homologs of Mdm33; however, analysis using HHPRED (Zimmermann et al., 2018), which searches for remote homology, identified the human IMM protein CCDC51 as the top hit (E-value 4.9E-10; see Materials and methods). Additionally, the AlphaFold2 predicted structures of Mdm33 and CCDC51 appear highly similar (Jumper et al., 2021) (Fig. S1 A). Notably, CCDC51, also called MITOK based on its suggested role as a mitochondrial K^+^ channel, is required for normal mitochondrial morphology (Paggio et al., 2019). CCDC51 is similar in length to Mdm33 (411 and 455 amino acids, respectively) and, like Mdm33, contains an N-terminal MTS and two TM domain segments that are likewise interspersed with predicted coiled-coil domains (Fig. 1 A).

In yeast ∆*mdm33* cells, mitochondria form extensive lariat-shaped rings and sheet-like lamellar structures (Klecker et al., 2015; Messerschmitt et al., 2003). To explore the mitochondrial morphology of CCDC51-depleted cells in more detail and perform a comparison to ∆*mdm33* yeast, we utilized CRISPR interference (CRISPRi) to transcriptionally repress and stably deplete endogenous CCDC51 from U2OS cells, a human osteosarcoma cell line. U2OS cells stably expressing the dCas9-KRAB transcriptional repressor (Le Vasseur et al., 2021) were transduced with a lentiviral plasmid expressing scrambled sgRNA or individual sgRNAs targeting the transcription start site of CCDC51. We generated two independent stable knockdown lines, each with near complete depletion of CCDC51 protein levels (Fig. 1 B). In each CCDC51-depleted cell line, mitochondria stained with the dye MitoTracker appeared to form lamellar, sheet-like structures in contrast to the tubular morphology observed in control cells (Fig. 1, C and D). MitoTracker also labeled mitochondria nonuniformly and was concentrated at the edge of the lamellar mitochondrial structures (Fig. 1 C). Additionally, we could occasionally observe small discontinuities that appeared as holes at the perimeter of lamellar structures (Fig. 1 C, arrows). Thus, consistent with observations in HeLa CCDC51 knockout (KO) cells (Paggio et al., 2019), CCDC51 is required for normal mitochondrial morphology.

To further characterize the mitochondrial morphology defect of CCDC51-depleted cells, we transfected cells with markers for the OMM (mCherry-OMP25), IMM (TIMM50-GFP), and matrix (mito-HaloTag labeled with JF646). In control cells, each marker uniformly labeled the mitochondrial membrane (Fig. 1 E, top panel). In contrast, each compartment marker displayed a unique localization pattern in CCDC51-depleted cells. The matrix marker formed lariat ring structures similar to those observed in ∆*mdm33* yeast cells (Messerschmitt et al., 2003) (Fig. 1 E, bottom panels). Conversely, the IMM and OMM markers labeled mitochondria more uniformly and appeared as lamellae that encapsulated the nonuniform matrix marker.

We then directly compared the mitochondrial morphology of CCDC51-depleted cells to yeast Δ*mdm33* cells. We chromosomally tagged wild-type and ∆*mdm33* yeast with functional markers of the OMM (Tom20-mCherry) and IMM (Pam17-EGFP) (Connor et al., 2023) and co-expressed a matrix marker (mito-TagBFP). As expected, mitochondrial morphology was altered in ∆*mdm33* cells, and the matrix marker formed hollow-appearing rings in many cells (Fig. 1 F). Strikingly, in a subset of cells where the mitochondria were flattened as in mammalian cells, the ringed matrix marker appeared encapsulated by both OMM and IMM markers (Fig. 1 F, bottom panels). These data indicate that the mitochondrial morphology of CCDC51-depleted cells is comparable with yeast ∆*mdm33* cells.

Previously, EM analysis of ∆*mdm33* cells revealed that the ring-like, hollow mitochondria visualized by fluorescence microscopy are connected to dilated regions that contain relatively normal-appearing cristae (Messerschmitt et al., 2003). To examine mitochondrial ultrastructure of CCDC51 CRISPRi cells, we performed thin-section EM analysis. Control cells predominantly showed tubular mitochondria with characteristic cristae (Fig. 1 G, left). In contrast, mitochondria in CCDC51-depleted cells were often swollen and enlarged with elongated cristae membranes (Fig. 1 G, middle). We also regularly observed mitochondria with internal ring structures, akin to Δ*mdm33* yeast cells (Fig. 1 G, right). As in yeast, these ring structures contained two membrane bilayers, and the cristae remained present in dilated portions of the organelle. These data further indicate that the mitochondrial morphology defects in the absence of Mdm33 and CCDC51 are comparable.

Next, to determine whether the two proteins are functionally orthologous, we examined whether expression of exogenous untagged human CCDC51 could complement the mitochondrial morphology defects of Δ*mdm33* yeast cells. Strikingly, in CCDC51-expressing ∆*mdm33* yeast cells, the mitochondrial morphology defects were significantly alleviated (Fig. 1, H and I). Thus, our data indicate that CCDC51 can help maintain mitochondrial morphology in the absence of Mdm33 in a cross-species expression system and suggest that the proteins are functionally conserved.

### Depletion of CCDC51 reduces the rate of mitochondrial fission

**Figure 2.**
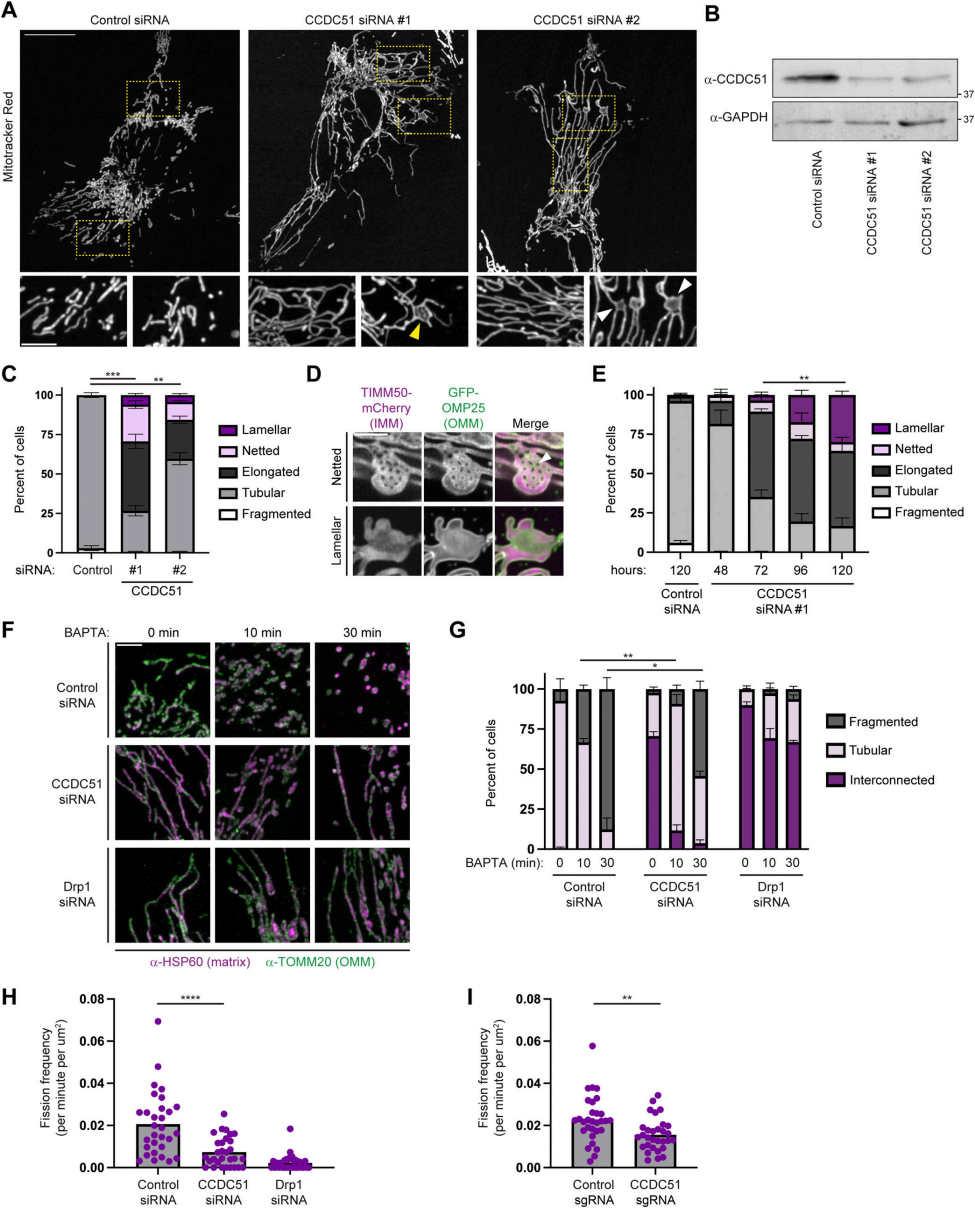
**Depletion of CCDC51 reduces the rate of mitochondrial fission. (A)** Representative maximum projection confocal images are shown of U2OS cells 72 h after treatment with the indicated siRNAs and stained with MitoTracker Red. Insets at the bottom correspond to the indicated dashed boxes. The yellow arrow marks a netted mitochondrion and white arrows mark lamellar mitochondria. **(B)** Western blot analysis with the indicated antibodies of lysate from cells treated as in A. **(C)** A graph of mitochondrial morphology characterization from the indicated siRNA-treated cells as in A and B. Data shown represent 100 cells per condition in each of three independent experiments, and bars indicate SEM. Asterisks (***P < 0.001; **P < 0.01) represent unpaired two-tailed *t* tests of tubular mitochondrial morphology. **(D)** Representative single-plane SoRa microscopy images of netted mitochondrial morphology (top) and lamellar mitochondrial morphology (bottom) of CCDC51 siRNA-treated cells expressing TIMM50-mCherry and GFP-OMP25. The arrow marks an example fenestra that spans both the OMM and IMM. **(E)** As in C at the indicated times after treatment with control siRNA or CCDC51 siRNA #1. Data shown represent 50–100 cells per condition in each of three independent experiments, and bars indicate SEM. Asterisks (**P < 0.01) represent an unpaired two-tailed *t* test of lamellar mitochondrial morphology. Corresponding representative images and western analysis are shown in Fig. S2, A and B. **(F)** Representative maximum intensity projection confocal images are shown of cells treated with the indicated siRNA and treated with 10 µM BAPTA-AM for the indicated times. See also Fig. S2 C for corresponding western analysis. **(G)** A graph of mitochondrial morphology characterization from cells as in F. Data shown represent 50–100 cells per condition in each of three independent experiments, and bars indicate SEM. Asterisks (**P < 0.01; *P < 0.03) represent unpaired two-tailed *t* tests of fragmented mitochondrial morphology. **(H and I)** A graph displaying the rate of mitochondrial fission for (H) individual cells treated with the indicated siRNA for 72 h or (I) the indicated CRISPRi cells. Cells were stained with MitoTracker Red and imaged for 5 min by single-plane confocal microscopy. For each cell, fission rates were determined in a 15 × 15-µm region of interest (ROI) of the cell periphery and were normalized to the 2D area of mitochondrial staining within the ROI. Data shown represent a total of 30 cells per condition acquired from two independent experiments. Asterisks (****P < 0.0001; **P < 0.01) represent unpaired two-tailed *t* tests. Scale bars: (A) 15 µm (5 µm in insets); (D) 5 µm; and (F) 4 µm. Source data are available for this figure: SourceData F2.

We reasoned that the lamellar mitochondrial morphology of CCDC51 CRISPRi cells could be a secondary consequence of long-term depletion of the protein. To examine the effect of acute depletion, we utilized RNAi to transiently knock down CCDC51. Cells were transfected with either a scrambled control siRNA or two independent siRNAs targeted against CCDC51. We then stained cells with MitoTracker and assessed mitochondrial morphology. As in the case of CRISPRi cells, we occasionally observed examples of lamellar mitochondria in cells depleted of CCDC51 by siRNA (Fig. 2, A–C; see Fig. 2 A white arrows). However, surprisingly, mitochondria were elongated and hyperfused in most cells, reminiscent of cells depleted of fission machinery such as Drp1 (Fig. 2, A and C) (Smirnova et al., 1998). We also commonly noticed the appearance of fenestrated “nets” of mitochondria (Fig. 2 A, see yellow arrow) that are similar to mitochondria found in yeast cells deficient for the Drp1 homolog Dnm1 (Bleazard et al., 1999; Sesaki and Jensen, 1999).

Given the differences in appearance between matrix and OMM markers in CCDC51 CRISPRi cells, we considered that the nets could represent an internal mitochondrial structure rather than reflect the overall morphology of the organelle. To examine the membrane organization of these structures in more detail, we transfected siRNA-treated cells with markers of the OMM (GFP-OMP25) and IMM (TIMM50-mCherry) and imaged them by super-resolution microscopy. We could readily visualize examples of net-like mitochondria with both tagged membrane proteins, indicating the holes span both membranes (Fig. 2 D). These data suggest that the mitochondrial nets present in CCDC51 knockdown cells are akin to those in yeast ∆*dnm1* cells.

**Figure S2.**
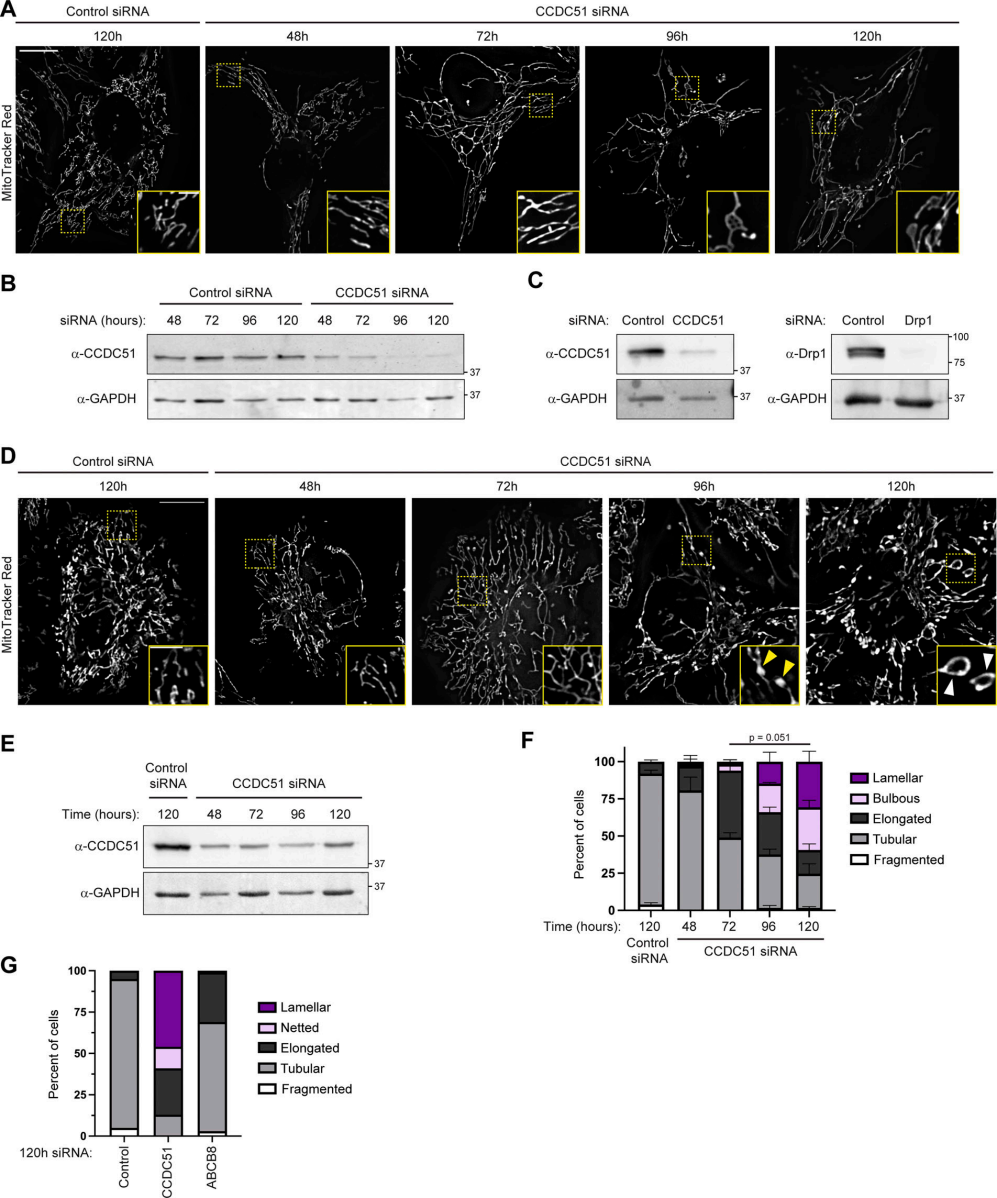
**Prolonged CCDC51 depletion leads to mitochondrial hyperfusion that precedes formation of lamellar mitochondria. (A)** Single-plane deconvolved images are shown of U2OS cells at the indicated times after treatment with the indicated siRNA oligonucleotides and stained with MitoTracker Red. Insets correspond to dashed boxes. Images correspond to quantification shown in Fig. 2 E. **(B)** Western blot analysis with the indicated antibodies of lysate from cells treated as in A and Fig. 2 E. **(C)** Western blot analysis with the indicated antibodies of lysate from cells corresponding to Fig. 2, F and G. **(D)** Single-plane deconvolved images are shown of HeLa cells at the indicated times after treatment with the indicated siRNA oligonucleotides and stained with MitoTracker Red. Insets correspond to dashed boxes. Yellow arrows mark mitochondrial bulbs and white arrows mark lamellar mitochondria. **(E)** Western blot analysis with the indicated antibodies of whole-cell lysate from cells treated as in D. **(F)** A graph is shown of mitochondrial morphology characterization from cells as in D and E. Data shown represents 50–100 cells per condition in each of three independent experiments, and bars indicate SEM. The P value shown represents an unpaired two-tailed *t* test of lamellar mitochondrial morphology. **(G)** A graph is shown of mitochondrial morphology characterization of cells treated with the indicated siRNA for 120 h. Data represent an average of results from two independent experimental replicates with at least 75 cells counted per experiment. Scale bars: (A) 15 µm (3 µm in inset); (D) 15 µm (5 µm in inset). Source data are available for this figure: SourceData FS2.

To next determine whether the lamellar mitochondrial appearance occurs due to prolonged CCDC51 depletion, we examined mitochondrial morphology at 24-h intervals after siRNA treatment. After 72 h, mitochondria were predominantly hyperfused with only occasional examples of nets and lamellae (Fig. 2 E; and Fig. S2, A and B). However, by 120 h, mitochondria frequently appeared lamellar (Fig. 2 E). We also examined the effect of CCDC51 siRNA treatment in HeLa cells. As in U2OS cells, mitochondria in HeLa cells were initially hyperfused and progressively formed lamellar structures during prolonged siRNA treatment (Fig. S2. D–F). Interestingly, we commonly observed cells whose mitochondria formed enlarged bulbs (Fig. S2, D–F, see yellow arrows), a phenotype associated with mtDNA accumulation in cells defective in mitochondrial fission (Ban-Ishihara et al., 2013). Together, these data indicate that acute depletion of CCDC51 causes mitochondrial elongation and nets in the short-term, followed by the formation of lamellar structures after prolonged knockdown.

The formation of hyperfused mitochondria in CCDC51-depleted cells suggests that loss of CCDC51 influences the relative frequency of mitochondrial fission dynamics. Previously, Mdm33 was proposed to aid in efficient mitochondrial fission, as Δ*mdm33* cells were resistant to sodium azide–induced Dnm1-dependent mitochondrial fragmentation (Klecker et al., 2015). To determine whether CCDC51-depleted cells are similarly resistant to stress-induced mitochondrial fission, we utilized the cell permeable Ca^2+^ chelator BAPTA-AM that has been previously shown to cause Drp1-dependent mitochondrial fragmentation (Friedman et al., 2011). U2OS cells depleted of CCDC51 or Drp1 by siRNA were mock-treated or incubated with BAPTA-AM for 10 and 30 min, fixed, and immunolabeled for markers of the OMM (TOMM20) and the matrix (HSP60). BAPTA-AM rapidly induced mitochondrial fission, and by 30 min, the mitochondrial network was completely fragmented in nearly all control cells, a phenotype that was prevented in cells depleted of Drp1 (Fig. 2, F and G). In contrast, while depletion of CCDC51 did not appear to block mitochondrial fission, it led to a delay in stress-induced mitochondrial fragmentation (Fig. 2, F and G), consistent with results in yeast ∆*mdm33* cells (Klecker et al., 2015).

Next, we asked whether the frequency of steady-state mitochondrial fission was affected in cells depleted of CCDC51. We performed live-cell imaging of MitoTracker-stained siRNA-treated cells and examined the frequency of mitochondrial fission events on an individual cell basis. As expected, Drp1 depletion nearly eliminated mitochondrial fission events (Fig. 2 H). In the absence of CCDC51, mitochondrial fission rates were significantly reduced, though not fully abolished (Fig. 2 H), consistent with our observations of resistance to stress-induced fission. We considered that the reduced rate of fission in cells depleted of CCDC51 by siRNA may be a transient phenotype. We therefore repeated our examination of mitochondrial dynamics in CCDC51 CRISPRi cells. Importantly, while more subtly affected than in acutely depleted cells, the rate of mitochondrial fission in CCDC51 CRISPRi cells was significantly reduced (∼32% less frequent) relative to control cells (Fig. 2 I). Together, these data indicate that reduced mitochondrial fission frequency contributes to the morphology defects of CCDC51-depleted cells.

One possibility that could explain these reduced mitochondrial fission rates is alteration in the factors involved in fission, including Drp1, OMM Drp1 adaptors such as MFF, or contacts between mitochondria and the ER (Kraus et al., 2021). However, in CCDC51 CRISPRi cells, Drp1 remained highly associated with mitochondria by immunolabeling (Fig. S1 B), consistent with observations in ∆*mdm33* yeast cells (Klecker et al., 2015). Additionally, MFF appeared enriched in puncta on the OMM in both control and CCDC51 CRISPRi cells (Fig. S1 C). ER–mitochondrial contacts and coordinated dynamics also did not appear markedly different in the absence of CCDC51, nor did they appear distinct at regions with lamellar mitochondria as compared with those with mitochondrial tubules (Fig. S1 D). We also performed time-lapse microscopy of CCDC51 CRISPRi cells that were transiently transfected with markers of the OMM (mCherry-OMP25) and matrix (mito-HaloTag), and consistent with MitoTracker-stained cells, we could observe persistent examples of mitochondrial fission and fusion, as well as the formation and dissolution of lamellar mitochondrial structures in relatively short time periods (Fig. S1 E; and Videos 1, 2, and 3). Altogether, our data indicate that mitochondrial fission is maintained but occurs less frequently in the absence of CCDC51.

**Video 1.** **Example of mitochondrial dynamics in a CCDC51 CRISPRi cell.** Single-plane time-lapse confocal microscopy images of a CCDC51 CRISPRi cell expressing mCherry-OMP25 (magenta) and mito-HaloTag/JF646 (green). Time is indicated as shown (sec = seconds). Scale bar = 3 µm. Still images of Video 1 are shown in Fig. S1 E.

**Video 2.** **Example of mitochondrial dynamics in a CCDC51 CRISPRi cell.** Single-plane time-lapse confocal microscopy images of a CCDC51 CRISPRi cell expressing mCherry-OMP25 (magenta) and mito-HaloTag/JF646 (green). Time is indicated as shown (sec = seconds). Scale bar = 3 µm. Still images of Video 2 are shown in Fig. S1 E.

**Video 3.** **Example of mitochondrial dynamics in a CCDC51 CRISPRi cell.** Single-plane time-lapse confocal microscopy images of a CCDC51 CRISPRi cell expressing mCherry-OMP25 (magenta) and mito-HaloTag/JF646 (green). Time is indicated as shown (sec = seconds). Scale bar = 3 µm. Still images of Video 3 are shown in Fig. S1 E.

### Overexpression of CCDC51 promotes Drp1-dependent mitochondrial fission

**Figure 3.**
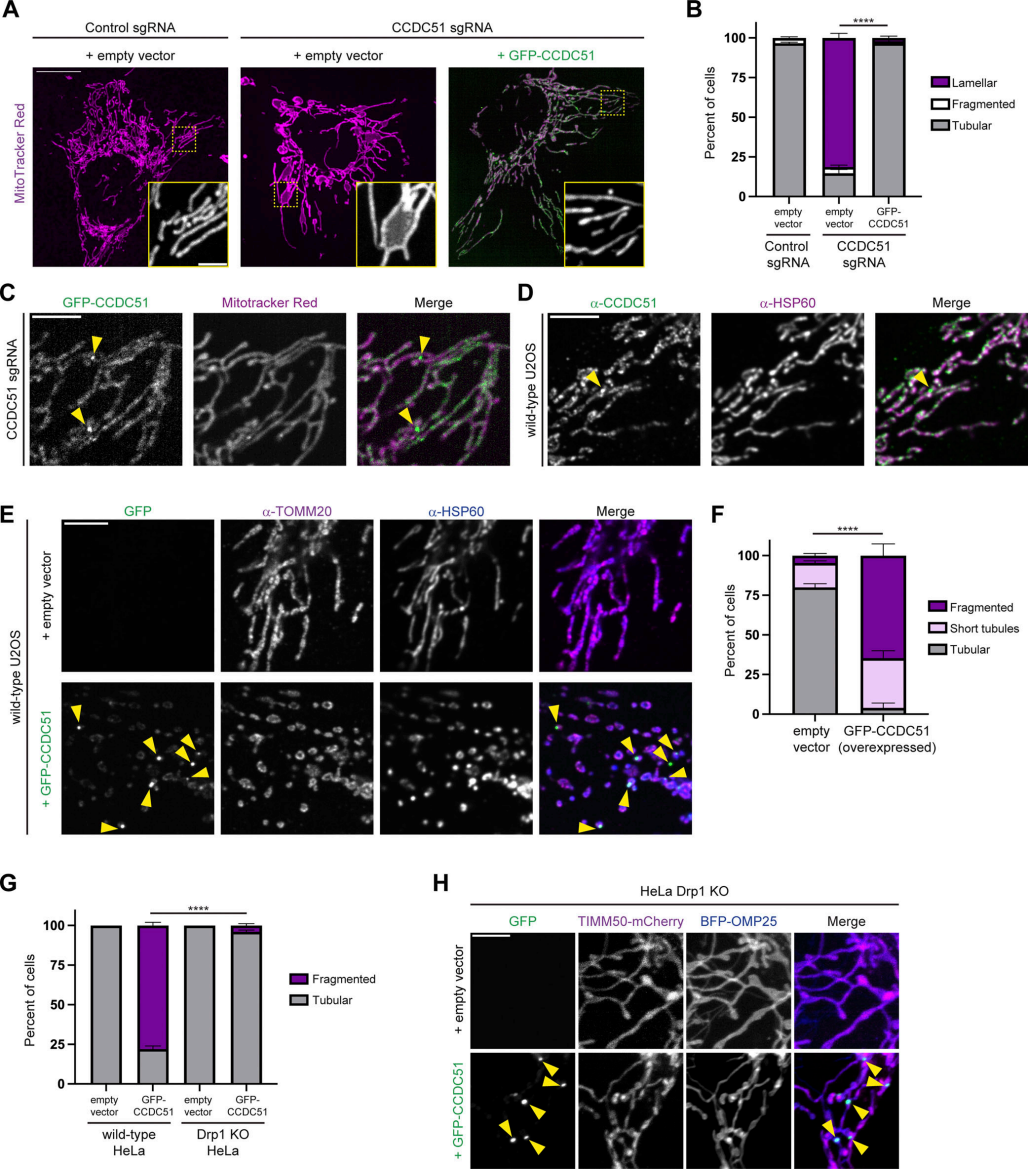
**Overexpression of CCDC51 promotes Drp1-dependent mitochondrial fission. (A)** Maximum projection confocal microscopy images of the indicated MitoTracker Red-stained U2OS CRISPRi cells expressing empty vector or low levels of GFP-CCDC51. Insets display MitoTracker staining corresponding to the dashed boxes. **(B)** A graph of mitochondrial morphology categorization from cells as in A. Data shown represent 50 cells per condition in each of three independent experiments, and bars indicate SEM. Asterisks (***P < 0.001) represent an unpaired two-tailed *t* test of tubular mitochondrial morphology. **(C)** A representative single-plane confocal microscopy image is shown of a CCDC51 CRISPRi cell, as in A, expressing GFP-CCDC51 and labeled with MitoTracker Red. Arrows mark sites of GFP-CCDC51 concentration at discrete foci along mitochondria. **(D)** A representative single-plane confocal microscopy image is shown of a U2OS cell fixed and immunolabeled for CCDC51 and HSP60. The arrow marks a site of CCDC51 enrichment. **(E)** Representative single-plane confocal images are shown of U2OS cells expressing empty vector (top) or high levels of GFP-CCDC51 (bottom) that were fixed and immunolabeled for TOMM20 and HSP60. Arrows mark examples of GFP-CCDC51 enrichment at discrete foci. **(F)** A graph is shown of mitochondrial morphology characterization from cells as in E. Data shown represents 50 cells per condition in each of three independent experiments, and bars indicate SEM. Asterisks (****P < 0.0001) represent an unpaired two-tailed *t* test of tubular mitochondrial morphology. **(G)** As in F for wild-type and Drp1 KO HeLa cells. Corresponding images are shown in Fig. S3 B. **(H)** Representative maximum intensity confocal images are shown of HeLa Drp1 KO cells expressing empty vector (top) or high levels of GFP-CCDC51 (bottom) and co-expressing TIMM50-mCherry and BFP-OMP25. Arrows mark sites of focal accumulation of GFP-CCDC51. See also Fig. S3 C for corresponding wild-type cells. Scale bars: (A) 15 µm (5 µm in inset); (C) 5 µm; (D) 5 µm; (E) 5 µm; and (H) 5 µm.

In addition to being required for normal mitochondrial morphology and dynamics, overexpression of Mdm33 causes Dnm1-dependent mitochondrial fragmentation in yeast (Klecker et al., 2015). We thus wanted to ask whether CCDC51 overexpression similarly causes mitochondrial fragmentation in human cells. We first generated an in-frame internally GFP-tagged version of CCDC51 that was functional as assessed by its ability to localize to mitochondria and fully rescue the mitochondrial morphology defects of CCDC51 CRISPRi cells when expressed at low levels (Fig. 3, A and B). In these cells, the GFP signal appeared in a patchy distribution throughout the mitochondrial network and occasionally enriched in discrete focal structures (Fig. 3 C, see arrows), similar to endogenous CCDC51 immunolabeled in wild-type cells (Fig. 3 D).

**Figure S3.**
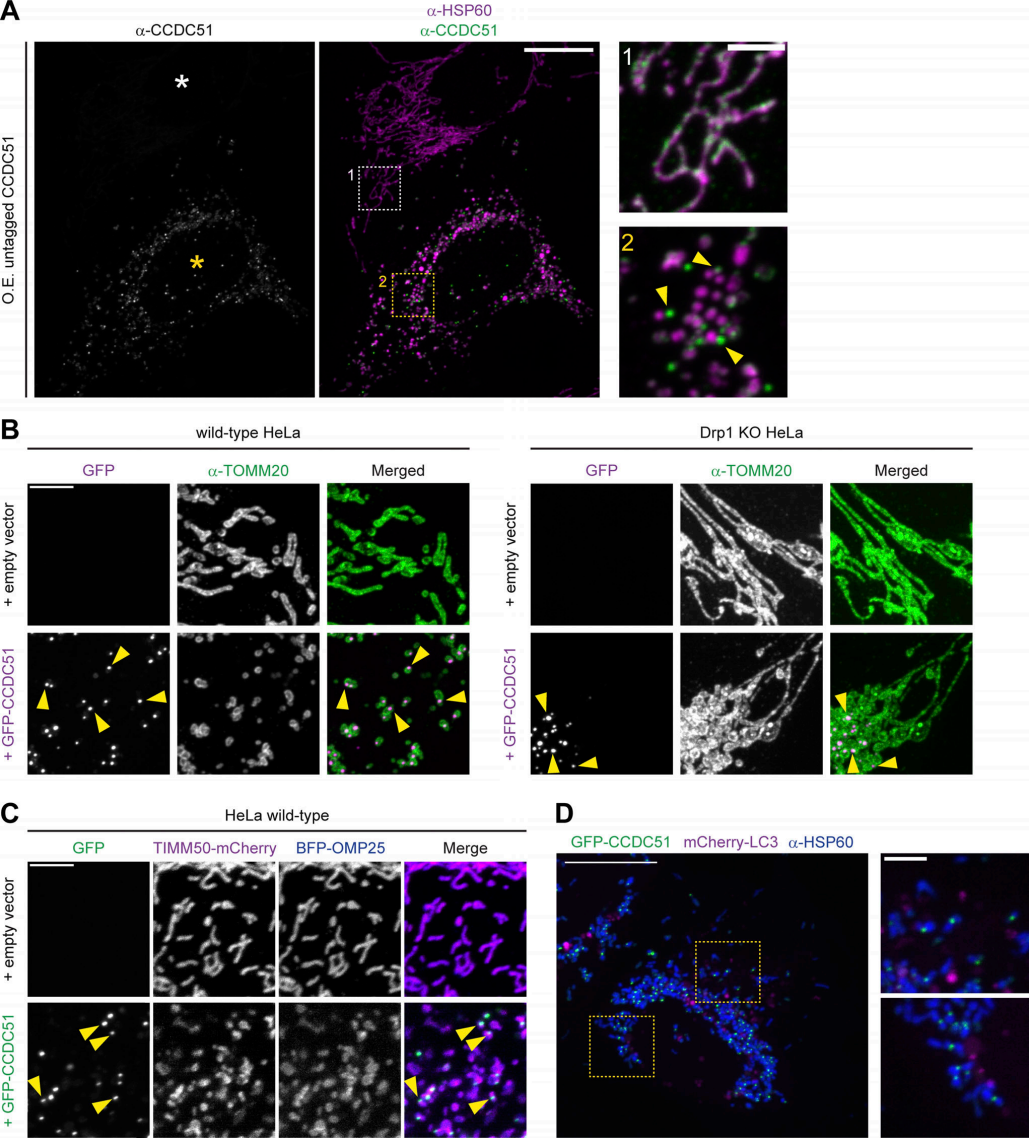
**CCDC51 overexpression leads to mitochondrial fragmentation and its accumulation at sites that do not co-localize with autophagy markers. (A)** Representative single-plane confocal images of U2OS cells transiently transfected with untagged CCDC51, fixed, and immunolabeled for HSP60 and CCDC51. The white asterisk marks a non-transfected cell with tubular mitochondrial morphology and endogenous distribution of CCDC51. The yellow asterisk marks a CCDC51-overexpressing cell with fragmented mitochondria in which CCDC51 concentrates at discrete sites (examples marked with yellow arrows). Numbered insets at right correspond to the dashed boxes shown at left. **(B)** Representative maximum projection confocal images of HeLa wild-type and Drp1 KO cells expressing empty vector (top) or overexpressing GFP-CCDC51 (bottom) that were fixed and immunolabeled for TOMM20. Images shown correspond to quantification shown in Fig. 3 G. Arrows mark examples of GFP-CCDC51 focal enrichment. **(C)** Representative maximum intensity projection confocal images are shown of wild-type HeLa cells expressing empty vector (top) or GFP-CCDC51 (bottom) and co-expressing TIMM50-mCherry and BFP-OMP25. Arrows mark examples of focal accumulation of GFP-CCDC51. See Fig. 3 H for corresponding images in HeLa Drp1 KO cells. **(D)** Representative maximum intensity projection confocal images are shown of a U2OS cell overexpressing GFP-CCDC51 and mCherry-LC3 that was fixed and immunolabeled for HSP60. Insets at right correspond to dashed boxes shown at left. Note that GFP-CCDC51 foci do not appear to associate with mCherry-LC3. Scale bars: (A and D) 15 µm (3 µm in insets); (B and C) 5 µm.

We then assessed the consequence of GFP-CCDC51 overexpression on the mitochondrial network. Wild-type U2OS cells were transfected with higher amounts of GFP-CCDC51, fixed, and immunolabeled with TOMM20 and HSP60 to assess mitochondrial morphology. In cells expressing high levels of GFP-CCDC51 (as established by an arbitrary fluorescence threshold), mitochondrial networks predominantly appeared fragmented (Fig. 3, E and F), consistent with published observations (Paggio et al., 2019). Interestingly, we also observed that higher levels of GFP-CCDC51 expression correlated with an increase in its localization to discrete focal structures (Fig. 3 E, see arrows). Importantly, the focal accumulation of CCDC51 was not a consequence of the GFP fusion, as the protein enriched at discrete sites in cells overexpressing untagged CCDC51 (Fig. S3 A).

Next, to determine whether overexpressed GFP-CCDC51 induced mitochondrial fragmentation in a Drp1-dependent manner, we utilized HeLa Drp1 KO cells (Oshima et al., 2021). As in U2OS cells, GFP-CCDC51 expression caused mitochondrial fragmentation in wild-type HeLa cells (Fig. 3 G; and Fig. S3, B and C). However, in the absence of Drp1, the mitochondrial network remained intact in nearly all GFP-CCDC51 overexpressing cells (Fig. 3 G and Fig. S3 B). Notably, overexpressed GFP-CCDC51 accumulated in discrete focal structures even in the absence of Drp1 (Fig. S3 B, see arrows). Given this localization pattern, we also considered whether CCDC51 overexpression could lead to IMM scission independently of the OMM. However, in Drp1 KO cells co-expressing GFP-CCDC51 and markers for the IMM (TIMM50-mCherry) and OMM (BFP-OMP25), the IMM remained continuous at the resolution of confocal microscopy, even at regions where GFP-CCDC51 enriched at foci (Fig. 3 H). Altogether, our data indicate that CCDC51 overexpression causes mitochondrial fragmentation in a Drp1-dependent manner, though CCDC51 is likely incapable of severing the IMM independently of Drp1.

### CCDC51 likely promotes normal mitochondrial morphology independently of K^+^ channel activity

**Figure 4.**
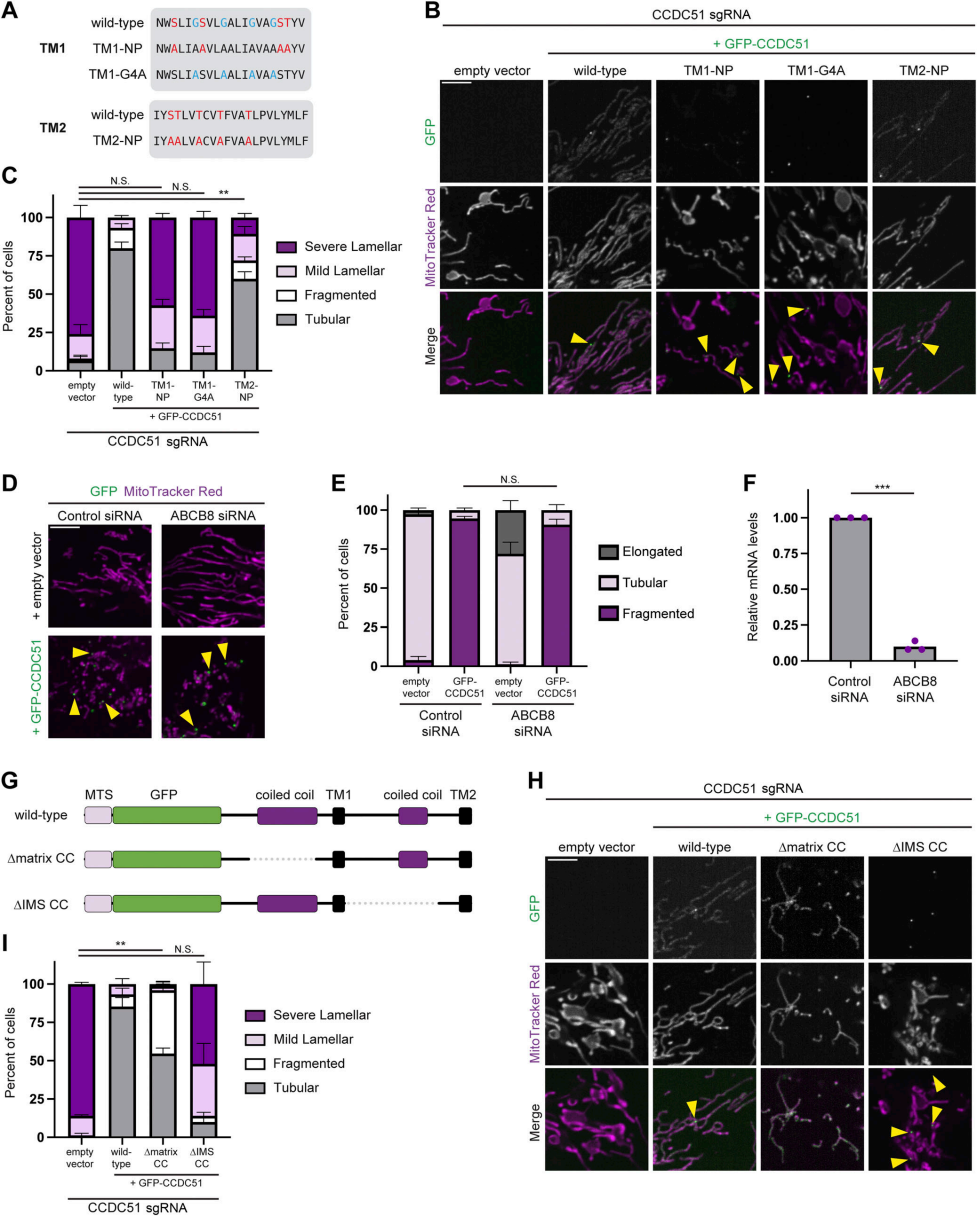
**CCDC51 likely promotes normal mitochondrial morphology independently of K** ^**+**^ **channel activity. (A)** A schematic displaying the amino acid sequence of the predicted TM domains (TM1 and TM2) of CCDC51. NP mutants of CCDC51 were generated (TM1-NP and TM2-NP) with the indicated point mutations in amino acids indicated in red. Mutations in the GxxxG motif (TM1-G4A) are indicated in blue. **(B)** Representative single-plane confocal images are shown of CCDC51 CRISPRi cells expressing an empty vector or low levels of the indicated GFP-CCDC51 constructs that were stained with MitoTracker Red. Arrows mark sites of focal accumulation of GFP-CCDC51. **(C)** A graph is shown of mitochondrial morphology characterization from cells as in B. Data shown represent 25 cells per condition in each of three independent experiments, and bars indicate SEM. Asterisks (**P < 0.01) represent an unpaired two-tailed *t* test of tubular mitochondrial morphology. N.S. indicates not statistically significant. **(D)** Representative single-plane confocal images of U2OS cells treated with the indicated siRNA for 72 h and expressing an empty vector (top) or high levels of GFP-CCDC51 (bottom) and stained with MitoTracker Red. Arrows mark examples of GFP-CCDC51 foci. **(E)** A graph is shown of mitochondrial morphology characterization from cells as in D. Data shown represent 25 cells per condition in each of three independent experiments, and bars indicate SEM. N.S. indicates not statistically significant results from an unpaired two-tailed *t* test of tubular mitochondrial morphology. **(F)** A graph is shown of relative mRNA levels as assessed by qRT-PCR of cells treated for 72 h with control- or ABCB8-targeting siRNA in three independent experiments. Asterisks (***P < 0.001) represent an unpaired two-tailed *t* test. **(G)** A schematic displaying GFP-CCDC51 variants with in-frame deletions of the matrix coiled-coil (∆matrix CC) or the IMS coiled-coil (∆IMS CC). **(H and I)** As in B and C for cells expressing the indicated GFP-CCDC51 forms indicated in G. Scale bars: 5 µm.

CCDC51 has been previously reported to act as the pore-forming subunit of an IMM K^*+*^ channel (Paggio et al., 2019), and we wondered whether this activity of the protein could contribute to its role in promoting normal mitochondrial morphology. Because CCDC51 lacks a highly conserved ion selectivity filter common to almost all other K^*+*^ channels (Mironenko et al., 2021; Paggio et al., 2019), we could not mutate these residues. However, we reasoned that the specific amino acid sequence within the TM domains of CCDC51, particularly polar amino acids, may be important for the passage of K^+^ ions. We therefore generated forms of GFP-CCDC51 with 4–5 serine or threonine residues in each TM domain mutated to nonpolar (NP) alanine residues (TM1-NP and TM2-NP, respectively; Fig. 4 A). Additionally, we noticed that the first TM domain of CCDC51 contains a glycine zipper motif (GxxxG) that is known to promote oligomerization and protein interactions of membrane-spanning proteins (Russ and Engelman, 2000), and we additionally generated an isoform with each of the four glycines of TM1 mutated to alanine (TM1-G4A; Fig. 4 A).

We transiently expressed wild-type or mutant forms of GFP-CCDC51 in CCDC51 CRISPRi cells, stained with MitoTracker, and imaged by confocal microscopy. Notably, in cells with similarly low expression levels, both wild-type GFP-CCDC51 and GFP-CCDC51(TM2-NP) demonstrated significant rescue of the mitochondrial morphology defects (Fig. 4, B and C). In contrast, both constructs with mutations in TM1 failed to rescue the lamellar mitochondrial morphology (Fig. 4, B and C). Interestingly, GFP-CCDC51 with mutations in TM1 (NP and G4A) localized to mitochondria but appeared highly concentrated in discrete foci (Fig. 4 B, see arrows). Together, these data indicate that while polar amino acids in TM2 are not critical for CCDC51 function, the amino acid sequence of TM1 is important for the ability of CCDC51 to maintain normal mitochondrial morphology.

Because mutations in each TM domain differentially affected rescue of mitochondrial morphology of CCDC51-depleted cells, we could not definitively conclude whether CCDC51 K^*+*^ channel activity was important for mitochondrial morphology and decided to address this by an orthogonal approach. Previously, CCDC51 was shown to act in a co-channel along with the IMM ATP-binding cassette protein ABCB8, which is proposed to regulate the K^+^ channel activity of CCDC51 (Paggio et al., 2019). We reasoned that if CCDC51 mediates mitochondrial morphology via K^+^ channel activity, ABCB8 depletion would lead to a similar mitochondrial morphology defect as CCDC51 knockdown and that mitochondrial fragmentation upon CCDC51 overexpression would occur in an ABCB8-dependent manner. While mitochondria in cells depleted of ABCB8 by siRNA were occasionally elongated relative to control cells, overexpression of GFP-CCDC51 led to its accumulation into focal structures and the efficient fragmentation of the mitochondrial network independently of ABCB8 (Fig. 4, D–F). We considered that the mild mitochondrial elongation of ABCB8-depleted cells could be an indication that CCDC51 knockdown is phenocopied by loss of ABCB8. However, even after longer-term siRNA treatment (120 h), ABCB8 depletion did not lead to the formation of lamellar mitochondria or mitochondrial nets (Fig. S2 G). Together, these data indicate that K^+^ channel activity of CCDC51 likely does not contribute to the role of the protein in promoting normal mitochondrial morphology or inducing mitochondrial fission when overexpressed.

As CCDC51 contains coiled-coil domains facing the mitochondrial matrix as well as the IMS, we considered that these domains could play functional roles in helping the protein to facilitate efficient mitochondrial fission. To address this, we transiently expressed GFP-CCDC51 variants with in-frame deletions of the matrix coiled-coil (∆matrix CC) or IMS coiled-coil (∆IMS CC) in CCDC51 CRISPRi cells (Fig. 4 G). In contrast to wild type GFP-CCDC51, the mitochondrial morphology defect of CCDC51-depleted cells was predominantly not rescued by GFP-CCDC51(∆IMS CC) (Fig. 4, H and I). This form of GFP-CCDC51 concentrated in discrete foci, similar to nonfunctional TM1 mutants (Fig. 4 H, see arrows). Cells expressing GFP-CCDC51(∆matrix CC) also exhibited distinct behavior from those expressing the wild-type construct. Notably, the lamellar mitochondrial morphology of CCDC51-depleted cells was completely alleviated, and mitochondrial networks were also more commonly fragmented (Fig. 4, H and I). In addition, the ∆matrix CC construct appeared to uniformly localize along mitochondria and was rarely observed to localize to enrich at discrete foci (Fig. 4 H). Together, our data suggest that both TM1 and the IMS coiled-coil domain of CCDC51 are important for the ability of the protein to distribute normally within mitochondria and to promote tubular mitochondrial morphology. Our data additionally reveal that the matrix coiled-coil domain is dispensable for CCDC51 to promote mitochondrial fission.

### CCDC51 spatially demarcates a subset of mitochondrial fission events

**Figure S4.**
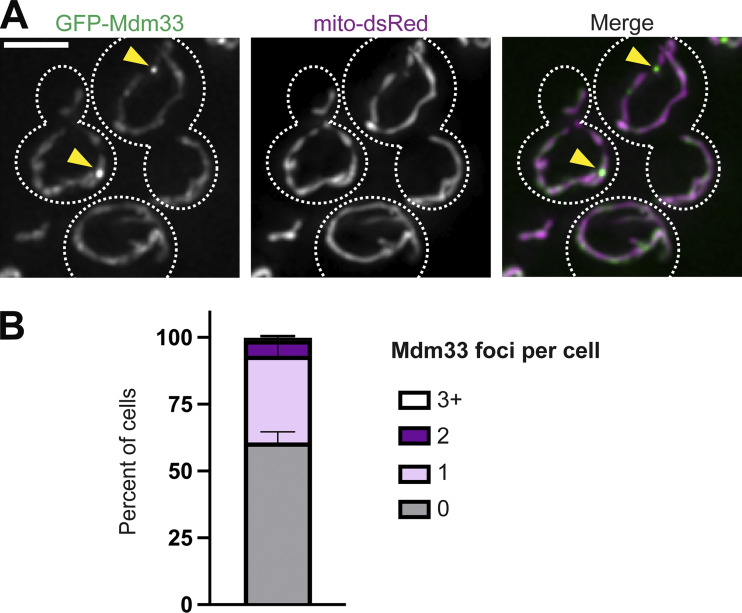
**Yeast GFP-Mdm33 concentrates at focal structures at steady state. (A)** A maximum intensity projection of a deconvolved fluorescence microscopy image is shown of wild-type yeast cells expressing GFP-Mdm33 from the endogenous chromosomal locus and co-expressing mito-dsRed. Dashed lines indicate cell outlines. Yellow arrows mark sites of GFP-Mdm33 focal accumulation. **(B)** A quantification of the indicated number of GFP-Mdm33 focal enrichments detected per cell as in A. Data shown represent 75 cells per condition in each of three independent experiments, and bars indicate SEM. Scale bars: 3 µm.

**Figure 5.**
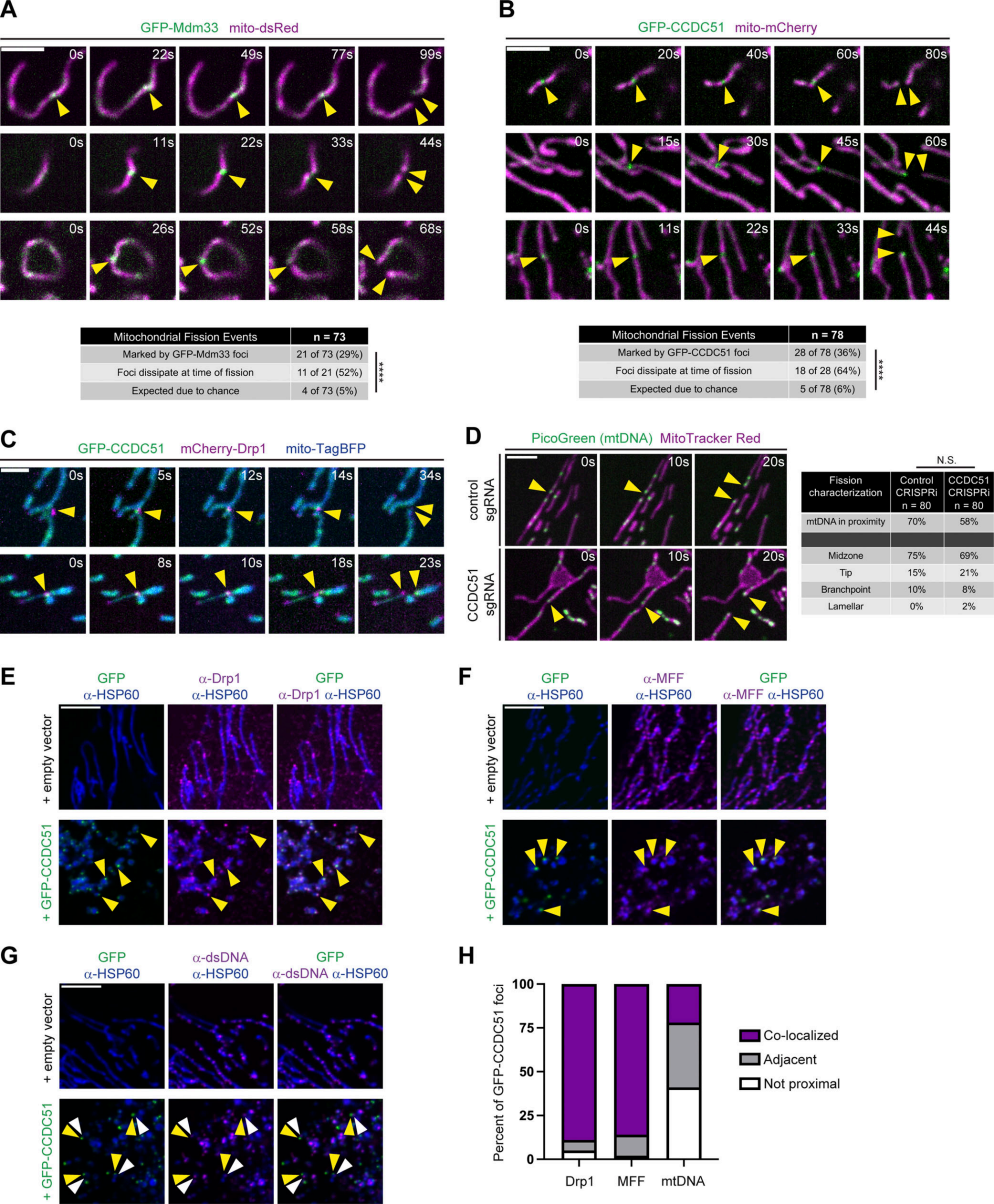
**CCDC51 spatially demarcates a subset of mitochondrial fission events. (A)** Single-plane confocal images are shown at the indicated time intervals (s = seconds) of yeast cells expressing endogenously-tagged GFP-Mdm33 and mito-dsRed. Arrows mark sites of GFP-Mdm33 focal enrichment relative to mitochondrial fission events. The table at bottom characterizes mitochondrial fission events marked by Mdm33 and their expected frequency due to chance (*n* = 73 fission events characterized). Asterisks (****P < 0.0001) represent a Fisher’s exact test. See also Videos 4 and 5. **(B)** Single-plane confocal images are shown as in A of U2OS cells expressing low levels of GFP-CCDC51 and mito-mCherry. Arrows mark sites of GFP-CCDC51 focal enrichment relative to mitochondrial fission events. See also Videos 6, 7, and 8. The table at bottom characterizes mitochondrial fission events marked by CCDC51 and their expected frequency due to chance (*n* = 78 fission events characterized). Asterisks (****P < 0.0001) represent a Fisher’s exact test. **(C)** Representative single-plane confocal images as in B of U2OS cells expressing GFP-CCDC51, mCherry-Drp1, and mito-TagBFP. Arrows mark examples of GFP-CCDC51 and mCherry-Drp1 co-localization at sites of mitochondrial fission. See also Videos 9 and 10. **(D)** Single-plane confocal images of the indicated CRISPRi cells stained with MitoTracker Red and PicoGreen (mtDNA). Arrows mark examples of mtDNA nucleoids that are spatially linked to mitochondrial fission events. The table at the right characterizes the frequency of mtDNA coordination with fission and the spatial distribution of fission events with relation to mitochondrial morphology (*n* = 80 fission events per condition). N.S. indicates not statistically significant results of a Fisher’s exact test of mtDNA proximity to fission events. **(E–G)** Single-plane confocal images are shown of U2OS cells expressing empty vector (top) or high levels of GFP-CCDC51 (bottom) that were fixed and immunolabeled for HSP60 and (E) Drp1, (F) MFF, or (G) dsDNA (mtDNA). Yellow arrows (E and F) mark examples of co-localization of GFP-CCDC51 foci with enrichments of Drp1 and MFF, respectively. White arrows in G mark examples of mtDNA nucleoids that localize adjacent to, but are not co-localized with, GFP-CCDC51 foci (yellow arrows). **(H)** A graph categorizing the spatial relationship between GFP-CCDC51 foci and enrichments of Drp1, MFF, and mtDNA from cells as in E–G. Data represent 100 GFP-CCDC51 foci characterized per condition. Scale bars: (A, B, and D) 3 µm; (C) 2 µm; and (E–G) 5 µm.

While both endogenous and GFP-labeled CCDC51 concentrate at discrete sites within mitochondria, and the prevalence of these enrichments correlates with increased fragmentation of the mitochondrial network, we considered that the focal enrichment of CCDC51 may not be related to the fission activity of the protein. Given the similarities between Mdm33 and CCDC51 and the relative ease of expressing yeast fusion constructs at endogenous expression levels, we wanted to determine if Mdm33 has a comparable sub-mitochondrial localization pattern to CCDC51 and if it could be spatially linked to mitochondrial fission events. We utilized a yeast strain previously established to express functional GFP-Mdm33 from the endogenous chromosomal locus (Klecker et al., 2015). As in the case of GFP-CCDC51, GFP-Mdm33 displayed a patchy distribution throughout the mitochondrial network and enriched in one to two discrete foci in almost half of cells at steady state (Fig. S4). We then performed live-cell microscopy to examine the dynamics of GFP-Mdm33 foci relative to mitochondrial fission events. Remarkably, nearly one-third of fission events (21 of 73; 29%) were marked by a GFP-Mdm33 foci (Fig. 5 A; and Videos 4 and 5). Importantly, the frequency of Mdm33 localization to a division site was significantly higher than expected due to chance based on the relatively sparse examples of GFP-Mdm33 enrichment at discrete foci along the dividing mitochondria (∼5% of the length of dividing mitochondrial tubules were covered by a foci) (Fig. 5 A). Additionally, GFP-Mdm33 was temporally linked to fission events, as the focal enrichment dissipated at the time of fission in about half of the events we captured (11 of 21 events; 52%) (Fig. 5 A). These data indicate that yeast Mdm33 is spatially and temporally linked to a subset of mitochondrial fission events.

**Video 4.** **Example of GFP-Mdm33 enrichment at a mitochondrial fission event in yeast.** Single-plane time-lapse confocal images of a wild-type yeast cell co-expressing GFP-Mdm33 (green) and mito-dsRed (magenta). Time is indicated as shown (sec = seconds). Scale bar = 3 µm. Still images of Video 4 are shown in Fig. 5 A.

**Video 5.** **Example of GFP-Mdm33 enrichment at a mitochondrial fission event in yeast.** Single-plane time-lapse confocal images of a wild-type yeast cell co-expressing GFP-Mdm33 (green) and mito-dsRed (magenta). Time is indicated as shown (sec = seconds). Scale bar = 3 µm. Still images of Video 5 are shown in Fig. 5 A.

Next, to determine if the spatial linkage to mitochondrial fission also occurs in human cells, we performed live-cell microscopy of wild-type U2OS cells transfected with GFP-CCDC51. We examined cells with low levels of GFP-CCDC51 expression and whose mitochondrial morphology remained tubular. Similar to our results in yeast, we found that 36% of fission events (28 of 78) were marked by GFP-CCDC51 foci, a value that is significantly higher than would be predicted due to chance (Fig. 5 B; and Videos 6, 7, and 8). Notably, the frequency of mitochondrial fission events marked by GFP-CCDC51 was similar to the reduction of the frequency of mitochondrial fission in CCDC51 CRISPRi cells (Fig. 2 I). Also, as we observed in yeast, the focal GFP-CCDC51 signal commonly dispersed simultaneously with completion of the fission event (18 of 28 events; 64%) (see Fig. 5 B top row). Further, GFP-CCDC51 foci could be observed to localize with mCherry-Drp1–marked fission events, indicating that the two proteins localize in proximity during fission (Fig. 5 C; and Videos 9 and 10). Together, our data indicate that both GFP-Mdm33 and GFP-CCDC51 are spatially and temporally associated with a subset of mitochondrial fission events.

**Video 6.** **Example of GFP-CCDC51 enrichment at a mitochondrial fission event in a U2OS cell.** Single-plane time-lapse confocal images of a U2OS cell co-expressing GFP-CCDC51 (green) and mito-mCherry (magenta). Time is indicated as shown (sec = seconds). Scale bar = 3 µm. Still images of Video 6 are shown in Fig. 5 B.

**Video 7.** **Example of GFP-CCDC51 enrichment at a mitochondrial fission event in a U2OS cell.** Single-plane time-lapse confocal images of a U2OS cell co-expressing GFP-CCDC51 (green) and mito-mCherry (magenta). Time is indicated as shown (sec = seconds). Scale bar = 3 µm. Still images of Video 7 are shown in Fig. 5 B.

**Video 8.** **Example of GFP-CCDC51 enrichment at a mitochondrial fission event in a U2OS cell.** Single-plane time-lapse confocal images of a U2OS cell co-expressing GFP-CCDC51 (green) and mito-mCherry (magenta). Time is indicated as shown (sec = seconds). Scale bar = 3 µm. Still images of Video 8 are shown in Fig. 5 B.

**Video 9.** **Example of GFP-CCDC51 and mCherry-Drp1 co-localization at a mitochondrial fission event in a U2OS cell.** Single-plane time-lapse confocal images of a U2OS cell co-expressing GFP-CCDC51 (green), mCherry-Drp1 (magenta), and mito-TagBFP (blue). Time is indicated as shown (sec = seconds). Scale bar = 2 µm. Still images of Video 9 are shown in Fig. 5 C.

**Video 10.** **Example of GFP-CCDC51 and mCherry-Drp1 co-localization at a mitochondrial fission event in a U2OS cell.** Single-plane time-lapse confocal images of a U2OS cell co-expressing GFP-CCDC51 (green), mCherry-Drp1 (magenta), and mito-TagBFP (blue). Time is indicated as shown (sec = seconds). Scale bar = 2 µm. Still images of Video 10 are shown in Fig. 5 C.

Like CCDC51, matrix-localized mtDNA nucleoids have also been observed to be coupled to a subset of mitochondrial fission events (Lewis et al., 2016). As CCDC51 has a matrix-localized coiled-coil domain, we reasoned that a function of CCDC51 could be to promote coupling of mtDNA nucleoids to mitochondrial fission sites. To examine this possibility, we first characterized the distribution of mtDNA in CCDC51 CRISPRi cells. While mtDNA localized to the rim of lamellar mitochondrial structures in cells depleted of CCDC51, consistent with the pattern of matrix protein localization, mtDNA appeared relatively normally distributed throughout the mitochondrial network (Fig. S1 F). Next, to specifically visualize mtDNA in relationship to mitochondrial fission events, we performed time-lapse microscopy of control and CCDC51 CRISPRi cells co-stained with MitoTracker and the mtDNA dye PicoGreen. We visualized a total of 80 mitochondrial fission events in each cell line and notably found that mtDNA was associated with a majority of fission events in both the presence and absence of CCDC51 (Fig. 5 D). We also examined these fission events for other characteristics, including their proximity to the tip of a mitochondrial tubule or sites of branching within the mitochondrial network, though did not notice an appreciable difference in cells depleted of CCDC51 (Fig. 5 D). Thus, while CCDC51 appears to mark and be responsible for a subset of steady-state fission events, there is not a clear distinguishing feature of events marked by CCDC51.

Our data indicate that while CCDC51 is not required for all mitochondrial fission events, its overexpression leads to its accumulation at discrete sites and to Drp1-dependent fragmentation of the mitochondrial network. While Mdm33 and CCDC51 expressed at close to endogenous levels occasionally enrich at sites that mark a subset of fission events, it is possible that the accumulation of CCDC51 during overexpression is unrelated to a positive role in fission. We therefore wanted to examine the relative localization of mitochondrial fission machinery to overexpressed GFP-CCDC51 foci. We transiently transfected wild-type U2OS cells with GFP-CCDC51 and fixed and immunolabeled for Drp1 or MFF. Remarkably, the vast majority of GFP-CCDC51 foci we examined were co-localized with Drp1 and MFF foci (Fig. 5, E and F, see yellow arrows; Fig. 5 H). These data indicate that overexpression of GFP-CCDC51 promotes the local recruitment of mitochondrial fission machinery to sites of CCDC51 concentration. We considered that overexpressed CCDC51 forms protein aggregates that are eliminated via mitochondrial fission related to mitophagy. However, focal accumulation of GFP-CCDC51 mutant constructs did not universally lead to mitochondrial fission (Fig. 4, B and H), and in cells overexpressing GFP-CCDC51, foci did not appear to co-localize with the autophagosome marker mCherry-LC3 (Gok et al., 2023) (Fig. S3 D). As an additional comparison, we examined the distribution of mtDNA relative to GFP-CCDC51 foci. While mtDNA occasionally localized adjacent to overexpressed GFP-CCDC51 foci, CCDC51 and mtDNA focal structures were substantially less co-localized than Drp1 or MFF (Fig. 5, G and H). Together, these data indicate that CCDC51 overexpression leads to the specific accumulation of Drp1 and its OMM receptor MFF at discrete and spatially linked subregions of the mitochondrial network.

### Conclusions

Our results reveal that Mdm33 and CCDC51 are functionally conserved positive effectors of Dnm1/Drp1–mediated mitochondrial division and that they spatially mark and facilitate a subset (approximately one-third) of basal fission events. What could their functional role be in fission and why do they mediate only a subset of events? While evidence suggests CCDC51 is a co-K^+^ channel along with ABCB8, our data indicate that this activity of the protein is likely not responsible for its role in fission. We alternatively considered, based on their conserved matrix-localized coiled-coil domains, that a function of Mdm33 and CCDC51 could be to couple mtDNA to sites of fission. However, our data indicate that mtDNA nucleoids remain associated with mitochondrial fission in the absence of CCDC51, and further, that overexpressed GFP-CCDC51 co-localizes with Drp1 and its receptor MFF, but infrequently with mtDNA. Another possible explanation comes from genetic interaction analysis of yeast Mdm33, which led Westermann and colleagues to propose a lipid homeostatic role of Mdm33 in mitochondrial dynamics (Klecker et al., 2015). Indeed, recent work suggests that local phospholipid remodeling at ER–mitochondrial contact sites may influence mitochondrial fission (Nguyen and Voeltz, 2022), and perhaps Mdm33 and CCDC51 could promote local phospholipid reorganization of the IMM at fission sites. A further, nonexclusive possibility is that Mdm33 and CCDC51 only participate in subsets of mitochondrial fission events by helping to coordinate or resolve cristae positioning with relation to fission sites. Such a function may help explain the unique mitochondrial morphology defects that arise from prolonged depletion of Mdm33 and CCDC51 compared with other mitochondrial fission machinery.

Insights into these questions may arise from future mechanistic studies into Mdm33 and CCDC51, as important questions remain with regards to their coordination with other fission machinery. Our structure–function analysis revealed that the IMS-localized coiled-coil, but not the matrix-localized coiled-coil, of CCDC51 is required for the ability of the protein to rescue mitochondrial morphology defects associated with its depletion. In addition, mutations in the first TM domain of CCDC51, including its glycine zipper motif, interfere with the function of the protein. An interesting possibility is that these IMS or TM domains, potentially through intermediary protein–protein interactions, promote coupling of CCDC51 with mitochondrial fission machinery on the OMM. In yeast, the IMS protein Mdi1/Atg44 is generally required for Dnm1 to complete mitochondrial fission (Connor et al., 2023; Furukawa et al., 2024). A potential relationship between Mdi1/Atg44 and Mdm33 and whether Mdi1 is functionally conserved in metazoans remain open questions. Regardless of its exact mechanistic and functional roles, the identification of an internal player in mitochondrial fission in human cells highlights an important new direction to explore how mitochondrial fission can be coordinated across two membrane bilayers.

## Materials and methods

### Cell culture

U2OS and HEK293T cells (kindly provided by Jodi Nunnari, Altos Labs, Redwood City, CA, USA) and HeLa wild type and Drp1 KO cells (kindly provided by Mariusz Karbowski, University of Maryland, College Park, MD, USA [Oshima et al., 2021]) were cultured in DMEM (D5796; Sigma-Aldrich) that was supplemented with 10% FBS (F0926; Sigma-Aldrich), 25 mM HEPES, and 1% penicillin/streptomycin (P4333; Sigma-Aldrich). All experiments using CRISPRi cells were performed on early passages (<10) after sorting. Cell lines were routinely tested for mycoplasma contamination.

### Plasmid construction and siRNA oligonucleotides

CCDC51 sgRNA plasmids were made by annealing the following oligonucleotide pairs into pU6-sgRNA-Ef1a-Puro-T2A-BFP linearized at the BstXI/BlpI sites (Horlbeck et al., 2016):

CCDC51 sgRNA #1 fwd: 5′-TTG​GGC​ACT​GCA​GGT​AGA​CAG​CAG​TTT​AAG​AGC-3′
CCDC51 sgRNA #1 rev: 5′-TTA​GCT​CTT​AAA​CTG​CTG​TCT​ACC​TGC​AGT​GCC​CAA​CAA​G-3′
CCDC51 sgRNA #2 fwd: 5′-TTG​GTC​GGG​CCA​CGC​CAG​GTA​CGG​TTT​AAG​AGC-3′
CCDC51 sgRNA #2 rev: 5′-TTA​GCT​CTT​AAA​CCG​TAC​CTG​GCG​TGG​CCC​GAC​CAA​CAA​G-3′.

GFP-CCDC51 was generated by cloning the MTS of CCDC51, AcGFP amplified from pAcGFP1-C1 (Clontech), and the CCDC51 ORF from human cDNA into the XhoI/NotI sites of pAcGFP1-N1 (Clontech) by Gibson Assembly. GFP-CCDC51 with TM mutations contain the following amino acid substitutions (TM1-NP = S204A, S208A, S219A, and T220A; TM1-G4A = G207A, G211A, G215A, and G218A; and TM2-NP = S389A, T390A, T393A, T396A, and T400A). GFP-CCDC51(∆matrix CC) contains an 8–amino acid Ser–Gly linker replacing amino acids 92–180 of CCDC51. GFP-CCDC51(∆IMS CC) contains a 12–amino acid Ser-Gly linker replacing amino acids 227–364 of CCDC51. All variants were generated from the parent GFP-CCDC51 plasmid by Gibson assembly. To overexpress untagged CCDC51, the CCDC51 coding sequence was amplified from GFP-CCDC51 and cloned into the XhoI/BamHI sites of pIRES2-dsRed2 (Clontech; a kind gift of Denise Marciano, UT Southwestern, Dallas, TX, USA).

GFP-OMP25 was a gift of Gia Voeltz (141150; Addgene). BFP-OMP25 and mCherry-OMP25 were generated by cloning the OMP25 cassette from GFP-OMP25 into the XhoI/BamHI sites of pTagBFP-C (Evrogen) and pmCherry-C1 (Clontech), respectively. TIMM50-GFP was generated by PCR amplifying the TIMM50 cassette from pLenti-mTIMM50-mRFP (a gift of Adam Hughes, University of Utah, Salt Lake City, UT, USA [Schuler et al., 2020, *Preprint*]) and cloning into the XhoI/BamHI sites of pAcGFP1-N1. TIMM50-mCherry was subsequently generated by digesting the mCherry cassette from pmCherry-N1 (Clontech) and cloning into the BamHI/NotI sites of TIMM50-GFP. To visualize the mitochondrial matrix, Halo-MTS (referred to as mito-HaloTag; a gift of Jin Wang, Baylor College of Medicine, Houston, TX, USA; 124315; Addgene), mito-mCherry (Kumar et al., 2024), or mito-TagBFP (Friedman et al., 2011) were used. To visualize ER morphology and dynamics, GFP-Sec61β was used (a gift of Gia Voeltz, University of Colorado Boulder, Boulder, CO, USA [Shibata et al., 2008]). To visualize Drp1 in live cells, mCherry-Drp1 (Friedman et al., 2011) was used. To visualize autophagy sites, mCherry-LC3 was used (Gok et al., 2023). pcDNA3 (Thermo Fisher Scientific) was used as the empty vector construct in all experiments.

For transient knockdowns, the following siRNAs were used: Negative control no. 2 (4390846; Thermo Fisher Scientific), CCDC51 oligo #1 (s36162; Thermo Fisher Scientific), CCDC51 oligo #2 (s36164; Thermo Fisher Scientific), DNM1L/Drp1 (s19560; Thermo Fisher Scientific), and ABCB8 (s22105; Thermo Fisher Scientific). CCDC51 oligo #1 was used in all experiments unless stated otherwise. Specific sequences are as follows:

CCDC51 #1 s36162: 5′-AGA​CUU​GGU​GGG​ACA​GAU​Att-3′
CCDC51 #2 s36164: 5′-GAC​UCA​ACG​AGG​UUC​GAG​Att-3′
Drp1 s19560: 5′-GAC​UUG​UCU​UCU​UCG​UAA​Att-3′
ABCB8 s22105: 5′-CGC​UUU​AAC​UGG​AAG​CUC​Utt-3′.

### U2OS CRISPRi cell generation

Stable CCDC51 CRISPRi cells were generated as previously described (Le Vasseur et al., 2021). Briefly, HEK293T cells were transiently transfected with standard lentiviral packaging plasmids, and the CCDC51 sgRNA plasmids described above. Viral supernatant was harvested and filtered through a 0.45-µm PES filter and added to U2OS dCas9 cells with 5.33 µg/ml polybrene. After infection and recovery, the top 50% brightest TagBFP-expressing cells were sorted by FACS. U2OS CRISPRi control sgRNA-expressing cells were described previously (Le Vasseur et al., 2021). CCDC51 sgRNA #2 was used in all experiments unless stated otherwise.

### Transient transfections

Approximately 200,000 cells were seeded per well of a 6-well dish and incubated overnight prior to transfection. Plasmid transfections were carried out with Lipofectamine 3000 (Thermo Fisher Scientific) according to the manufacturer’s instructions for 5–6 h. For standard siRNA treatments, transfections were performed in 6-well plates with Lipofectamine RNAiMAX (Thermo Fisher Scientific) and 20 nM RNAi oligonucleotides according to the manufacturer’s instructions. Cells were incubated for ∼24 h, passaged 1:2, grown an additional 24 h, and transfected again for 5 h with Lipofectamine 3000 with 20 nM RNAi oligonucleotides and any additional plasmids, where indicated. Cells were then passaged into glass-bottom microscope dishes or standard growth dishes and incubated for ∼24 h prior to imaging or harvest for western analysis. For knockdown time-course experiments, cells were treated as above and were imaged or harvested for lysate generation after a single transfection (48 and 72 h) or after two rounds of transfection (96 and 120 h).

### Mitochondrial labeling and drug treatments

To visualize mitochondria with MitoTracker, cells were stained with 25 nM MitoTracker CMXRos (M7512; Thermo Fisher Scientific) in DMEM for 30–60 min at 37°C, washed twice, and imaged live. To label cells transfected with mito-HaloTag, cells were stained with 1 µM Janelia Fluor 646 (Promega) for 30 min at 37°C and washed two times in growth media prior to imaging. To visualize mtDNA for live-cell time-lapse imaging, cells were stained with PicoGreen (P7589; Thermo Fisher Scientific) 1:1,000 in cell media for 1 h and washed twice prior to imaging. For BAPTA-AM treatment, cells were incubated with 10 µM BAPTA-AM (196419; Calbiochem) in DMEM for 10 or 30 min prior to processing for immunofluorescence as described below.

### Immunofluorescence

Cells cultured on glass-bottom dishes were fixed in 4% paraformaldehyde in PBS for 15 min at room temperature. Cells were permeabilized for 5 min with 0.1% Triton X-100 in PBS, rinsed with PBS, and incubated in blocking solution for 30 min (PBS supplemented with 0.1% Triton X-100 and 10% FBS). Cells were then incubated with the following primary antibodies, where indicated, in blocking solution for 30–60 min: rabbit anti-CCDC51 (20465-1-AP; Proteintech), mouse anti-TOMM20 (ab56783; Abcam), mouse anti-HSP60 (66041-1-Ig; Proteintech), rabbit anti-HSP60 (15282-1-AP; Proteintech), mouse anti-DLP1 (Drp1) (611112; BD Biosciences), rabbit anti-MFF (17090-1-AP; Proteintech), or mouse anti-dsDNA (ab27156; Abcam). Cells were washed several times with PBS and subsequently incubated with one or more of the following antibodies, as appropriate, in blocking solution for 30–60 min: donkey anti-rabbit Alexa Fluor 488 (A-21206; Thermo Fisher Scientific), donkey anti-mouse Alexa Fluor 555 (A-31570; Thermo Fisher Scientific), donkey anti-mouse Alexa Fluor 647 (A-31571; Thermo Fisher Scientific), or donkey anti-rabbit Alexa Fluor 647 Plus (A32795; Thermo Fisher Scientific). Cells were washed several times with PBS prior to imaging.

### qPCR

RNA was extracted from U2OS cells treated with control or ABCB8-targeting siRNA using the Monarch Total RNA Miniprep Kit (NEB) according to the manufacturer’s instructions. 1 mg of RNA was used to make cDNA using the iScript Reverse Transcription Supermix (BioRad). qPCR was performed with cDNA and the SsoAdvanced Universal SYBR Green Supermix (BioRad) on a CFX96 Real-Time System (BioRad). Predesigned KiCqStart SYBR Green primers targeting exons 2–3 of ABCB8 (NM_007188 H_ABCB8_3; Sigma-Aldrich) were used, and all samples were normalized to cyclophilin A (*CYPA*) as a reference gene.

### Whole-cell lysate preparation and western blots

Trypsinized cell pellets were washed and lysed with freshly prepared 1x RIPA buffer (50 mM Tris-HCl, pH 7.5, 150 mM NaCl, 1% Na-deoxycholate, 0.1% SDS, 1% NP-40, and 1 mM EDTA) containing 1x protease inhibitor cocktail (539131; Sigma-Aldrich) and incubated on ice for 30 min. Lysates were subjected to centrifugation (13,000 *g*, 4°C, 10 min), supernatant was collected, and protein concentration was determined by a Bradford or BCA assay and normalized between samples. 6x Laemmli sample buffer (6% SDS, 21.6% glycerol, 0.18 M pH 6 Tris-HCl, and bromophenol blue) supplemented with 10% β-mercaptoethanol was added to a final concentration of 1x. Samples were incubated at 95°C for 5 min and lysates were resolved by electrophoresis on Tris-glycine polyacrylamide gels. Protein was transferred to 0.45-µm PVDF membranes and immunoblotted with the following primary antibodies, where indicated: rabbit anti-CCDC51 (1:2,500; 20465-1-AP; Proteintech), mouse anti-GAPDH (1:25,000; 60004-1-Ig; Proteintech), rabbit anti-GAPDH (1:25,000; 10494-1-AP; Proteintech), or mouse anti-DLP1 (1:1,000; 611112; BD Biosciences). The following secondary antibodies were used for detection: goat anti-rabbit DyLight 800 (1:10,000; SA5-35571; Thermo Fisher Scientific), goat anti-mouse DyLight 800 (1:10,000; SA5-35521; Thermo Fisher Scientific), goat anti-mouse DyLight 680 (1:10,000; SA5-35518; Thermo Fisher Scientific), or goat anti-rabbit DyLight 680 (1:10,000; 35568; Thermo Fisher Scientific). Images were acquired by a LI-COR Odyssey Infrared Imaging System or a BioRad ChemiDoc Imaging System, and linear adjustments to images were performed using Adobe Photoshop.

### Yeast strain generation and cell growth

*Saccharomyces cerevisiae* strains were constructed in the W303 genetic background (*ade2-1*; *leu2-3*; *his3-11, 15*; *trp1-1*; *ura3-1*; and *can1-100*). Routine cell growth was performed in YPD (1% yeast extract, 2% peptone, and 2% glucose) or synthetic complete dextrose (SCD; 2% glucose, 0.7% yeast nitrogen base, and amino acids). Strains expressing chromosomally tagged Pam17-EGFP, Tom20-EGFP, and Tom20-mCherry were described previously (Connor et al., 2023). *MDM33* deletion was performed by PCR-based homologous recombination and the entire ORF was replaced with the HIS or NatMX6 cassettes from pFA6a series plasmids using lithium acetate transformation.

A strain expressing GFP-Mdm33 integrated at the endogenous locus was a kind gift of Jodi Nunnari (Klecker et al., 2015). To visualize the mitochondrial matrix in yeast cells, pRS304 mito-TagBFP, pRS304 mito-dsRed, and pYX142-DsRed were used (Connor et al., 2023; Friedman et al., 2011). To express human CCDC51 in yeast cells, pRS306 GalLprom-CCDC51-ADH1term was generated by Gibson assembly. This plasmid was linearized and integrated at the *ura3-1* locus. To control expression of CCDC51 with estradiol, pAGL (Veatch et al., 2009) was linearized and integrated at the *leu2-3* locus. CCDC51 expression was controlled by supplementing growth media with 20 nM β-estradiol (3301; Calbiochem).

For imaging, cells were grown at 30°C in SCD growth media to exponential phase (where indicated, in the presence of estradiol), concentrated, and plated on a 3% agarose pad in SCD media on depression microscope slides. Cells were subsequently imaged as described below.

### Epifluorescence and confocal microscopy and analysis

All U2OS and HeLa imaging experiments were performed on cells adhered to glass-bottom dishes (D35-14-1.5-N; CellVis or P35G-1.5-1-4C; MatTek), and all live imaging of human cells was performed at 37°C. Epifluorescence microscopy (images in Fig. S2 and Fig. S4) was performed with a Nikon Eclipse Ti-inverted epifluorescence microscope equipped with a Hamamatsu Orca-Fusion sCMOS camera, a Nikon 100× 1.45 NA objective, and an environmental control chamber. Z-series images were acquired with Nikon Elements software with a 0.2-µm step size, and all images were further deconvolved using AutoQuantX 3.1 (10 iterations, blind deconvolution, and low noise). Confocal microscopy (all other figures) was performed with a Nikon Spinning Disk Confocal Microscope equipped with a Yokagawa CSU-W1, a Hamamatsu Orca-Fusion sCMOS camera, 100× 1.45 NA objective, and an environmental control chamber. Images were acquired with the Nikon Elements software using the standard spinning disk module or, where indicated, a super-resolution SoRa module. Confocal z-series were acquired with a 0.2-µm step size, except where noted below. Acquisition settings were kept the same for non-transfected control cells or empty vector transfected cells within the same experiments. Linear adjustments to all images and all subsequent analyses were performed using ImageJ/Fiji.

#### Mitochondrial morphology assessment

In all cases, sample identity was blinded before assessment, and cells were manually categorized as indicated in figures and legends. In the case of cells with mixed mitochondrial morphologies, cells were categorized with the more prominent morphology. For yeast samples, multiple fields of view were acquired per experiment and no more than ∼25 cells were assessed per field of view per experiment.

#### Mitochondrial fission frequency assessment

To determine the frequency of mitochondrial fission, 5-min single-plane time-lapse movies were taken at ∼10-s intervals of MitoTracker-stained cells. A 15 × 15-µm^2^ well-resolved ROI in the cell periphery was analyzed for individual cells blind to sample identity, and the number of fission events was determined. A binary mask of mitochondrial staining was then generated for each ROI using the manual Threshold feature of ImageJ to determine the 2D area of mitochondrial staining, and the number of fission events per µm^2^ of mitochondria per cell was determined.

#### The relationship between Mdm33 and CCDC51 foci and mitochondrial fission events

To assess GFP-Mdm33 foci dynamics, 2-min time-lapse movies were taken at ∼5-s intervals with three z-planes centered on the midplane of yeast cells with a 0.3- or 0.4-µm step size. To assess whether GFP-Mdm33 foci localize to sites of mitochondrial division, maximum intensity projections were generated, and fission events were identified blind to the presence of GFP-Mdm33 foci. Single-plane images were then evaluated manually for the presence and spatial relationship to GFP-Mdm33 focal accumulations.

To assess GFP-CCDC51 foci relative to mitochondrial fission events and GFP-CCDC51 foci relative to mCherry-Drp1, 2-min single-plane time-lapse movies were taken at ∼1-s intervals. Cells were chosen for analysis only if GFP-CCDC51 expression was below an arbitrary fluorescence intensity threshold and mitochondrial morphology appeared tubular. Fission events were identified blind to the presence of GFP signal and then were evaluated manually for the presence and spatial relationship to GFP-CCDC51 and/or mCherry-Drp1.

To determine the probability that a GFP-CCDC51 or a GFP-Mdm33 foci would appear at a division site by chance, the length of the dividing mitochondria tubule was measured at the time point immediately preceding fission in a single plane of view. The length of the mitochondrial tubule that was covered by any discrete GFP foci was then computed and the probability was determined as the ratio between the two.

#### Assessment of mtDNA spatial relationship to fission events

To determine the spatial relationship of mtDNA to fission events, CRISPRi cells were co-stained with PicoGreen and MitoTracker Red and imaged for 5 min at ∼10-s intervals. Fission events were identified blind to the presence of PicoGreen signal and were manually assessed for proximity (defined as less than ∼1 µm) of PicoGreen-stained foci to the fission sites. To characterize mitochondrial fission events in relationship to mitochondrial morphology, midzone and peripheral (tip) fission events were categorized as previously described (Kleele et al., 2021). Branch points were defined as a three-way intersection of mitochondrial tubules, and lamellar fissions occurred in proximity to aberrantly shaped mitochondria in CCDC51 CRISPRi cells.

#### The relationship between overexpressed GFP-CCDC51 foci and Drp1, MFF, and mtDNA

To examine the proximity of GFP-CCDC51 foci to mitochondrial fission markers and mtDNA, U2OS cells overexpressing GFP-CCDC51 were fixed and immunolabeled for HSP60 as well as MFF, Drp1, or dsDNA (mtDNA). Cells were chosen for analysis only if GFP-CCDC51 expression was within an arbitrary fluorescence intensity threshold where mitochondrial morphology appeared fragmented, but the mitochondrial networks were not aggregated or collapsed. GFP-CCDC51 focal structures were identified blind to the presence of other markers before assessing their proximity. Foci were considered co-localized if they displayed more than ∼75% overlap. Foci were considered adjacent when they displayed less than ∼25% overlap or were immediately adjacent. A total of 100 foci were counted per condition in each of two independent experiments.

### EM

To assess mitochondrial ultrastructure by EM, 75,000 cells were plated onto glass-bottom dishes (MatTek), incubated overnight, and fixed with 2.5% (vol/vol) glutaraldehyde in 0.1 M sodium cacodylate buffer before submitting to the UTSW Electron Microscopy Core Facility for processing as described previously (Gok et al., 2023). After five rinses in 0.1 M sodium cacodylate buffer, cells were postfixed in 1% osmium tetroxide and 0.8 % K_3_[Fe(CN_6_)] in 0.1 M sodium cacodylate buffer for 1 h at 4°C. Cells were rinsed with water and en bloc stained with 2% aqueous uranyl acetate overnight at 4°C. After five rinses with water, specimens were dehydrated with increasing concentration of ethanol at 4°C, infiltrated with Embed-812 resin, and polymerized in a 60°C oven overnight. Embed-812 discs were removed from MatTek plastic housing by submerging the dish in liquid nitrogen. Pieces of the disc were glued to blanks with super glue and blocks were sectioned with a diamond knife (Diatome) on a Leica Ultracut UCT (7) ultramicrotome (Leica Microsystems) and collected onto copper grids and poststained with 2% uranyl acetate in water and lead citrate. Images were acquired on a JEM-1400 Plus transmission electron microscope equipped with a LaB_6_ source operated at 120 kV using an AMT-BioSprint 16M CCD camera.

### Bioinformatic analysis

To find putative metazoan homologs to Mdm33, HHPRED (Zimmermann et al., 2018) was used. An initial search of *S. cerevisiae* Mdm33 against the *Homo sapiens* proteome with default settings identified CCDC51 as the top hit (E-value 2.7E-9 with next closest E-value 0.22). A repeat analysis with “global realignment” again identified CCDC51 (E-value 4.9E-10) as the top hit. An inverse query of the protein sequence of CCDC51 against the proteomes of *S. cerevisiae* and *Schizosaccharomyces pombe* with default settings also identified Mdm33/She9 (E-values 8.2E-4 and 2.3E-4, respectively). TM domain segments of Mdm33 and CCDC51 were identified by Phobius. Coiled-coil domains in Mdm33 and CCDC51 were identified by consensus of multiple coiled-coil prediction programs.

### Statistical analysis

Statistical analysis was performed as indicated in legends with GraphPad Prism 9.5.1. Comparisons of the relationship of GFP-CCDC51 or GFP-Mdm33 foci to fission sites versus the predicted frequency due to chance (Fig. 5, A and B) were tested by Fisher’s exact test. Comparisons of the relationship of mtDNA foci to fission sites in control versus CCDC51-depleted cells (Fig. 5 D) were tested by Fisher’s exact test. Comparisons of mitochondrial morphology (all other figures) were performed by two-tailed Student’s *t* test of the indicated mitochondrial morphology category. In all such cases, data distribution was assumed to be normal, but this was not formally tested.

### Online supplemental material

Fig. S1 shows additional characterization of CCDC51 CRISPRi cells. Fig. S2 shows a temporal analysis of CCDC51 depletion. Fig. S3 shows analysis of cells with overexpressed CCDC51. Fig. S4 shows a characterization of yeast cells expressing GFP-Mdm33. Videos 1, 2, and 3 show examples of mitochondrial dynamics in CCDC51 CRISPRi cells. Videos 4 and 5 show examples of GFP-Mdm33 enrichment at mitochondrial fission sites in yeast. Videos 6, 7, and 8 show examples of GFP-CCDC51 enrichment at mitochondrial fission sites in U2OS cells. Videos 9 and 10 show examples of GFP-CCDC51 and mCherry-Drp1 localization to mitochondrial fission sites in U2OS cells.

## Supplementary Material

SourceData F1 is the source file for Fig. 1.

SourceData F2 is the source file for Fig. 2.

SourceData FS2 is the source file for Fig. S2.

## Data Availability

All the data underlying this study are available in the published article and its online supplemental material.
