# Supplementary figures and images for: Functionally conserved inner mitochondrial membrane proteins CCDC51 and Mdm33 demarcate a subset of fission events
Source: J Cell Biol. 2024 Dec 24;224(3):e202403140. doi: 10.1083/jcb.202403140 (PMC11668171; doi:10.1083/jcb.202403140)

Source Data F1

Fig. 1B

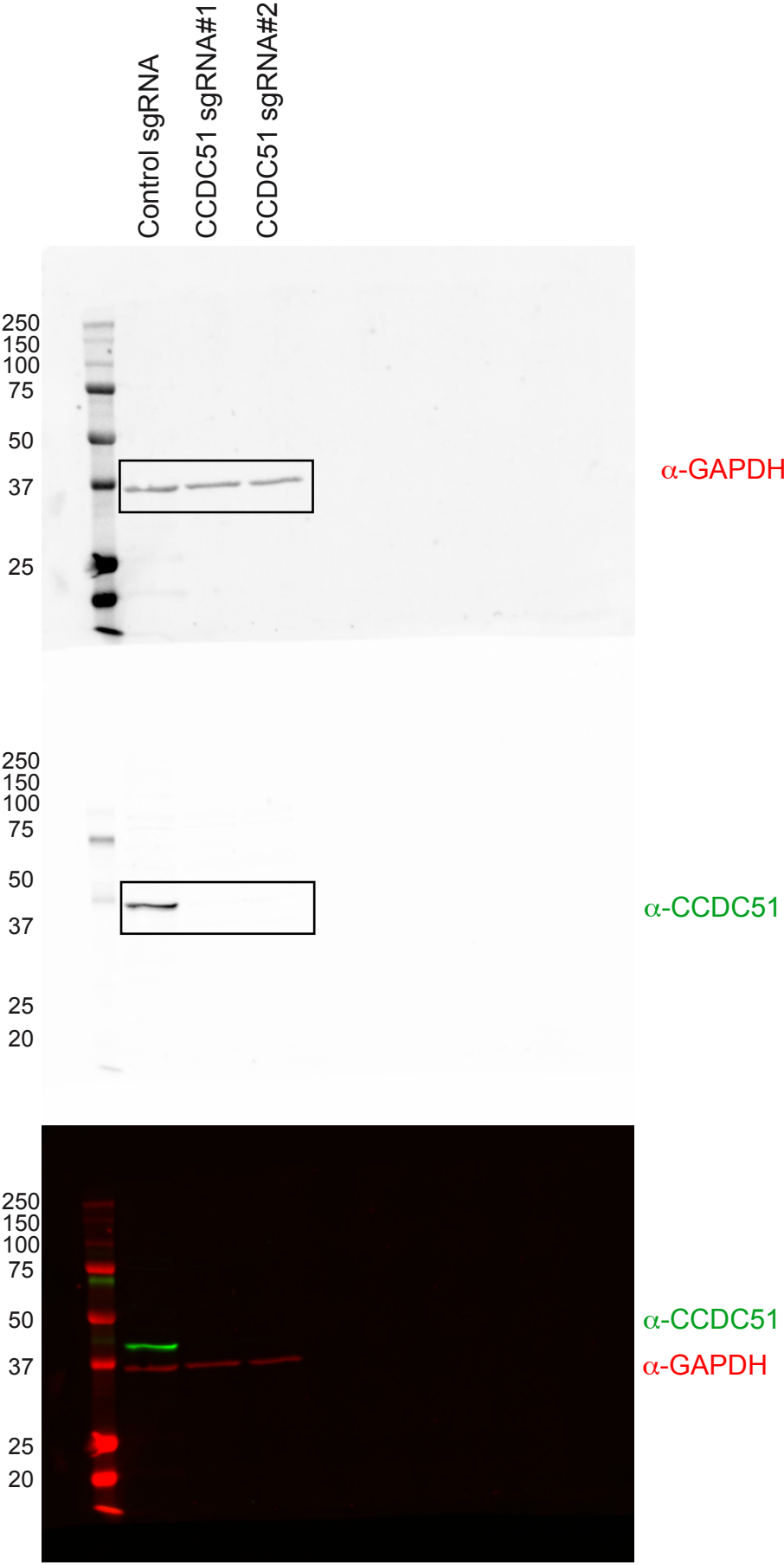

Supplement: SourceData F1 — is the source file for Fig. 1. [file jcb_202403140_sourcedataf1.pdf]

## Source Data F2

Fig. 2B

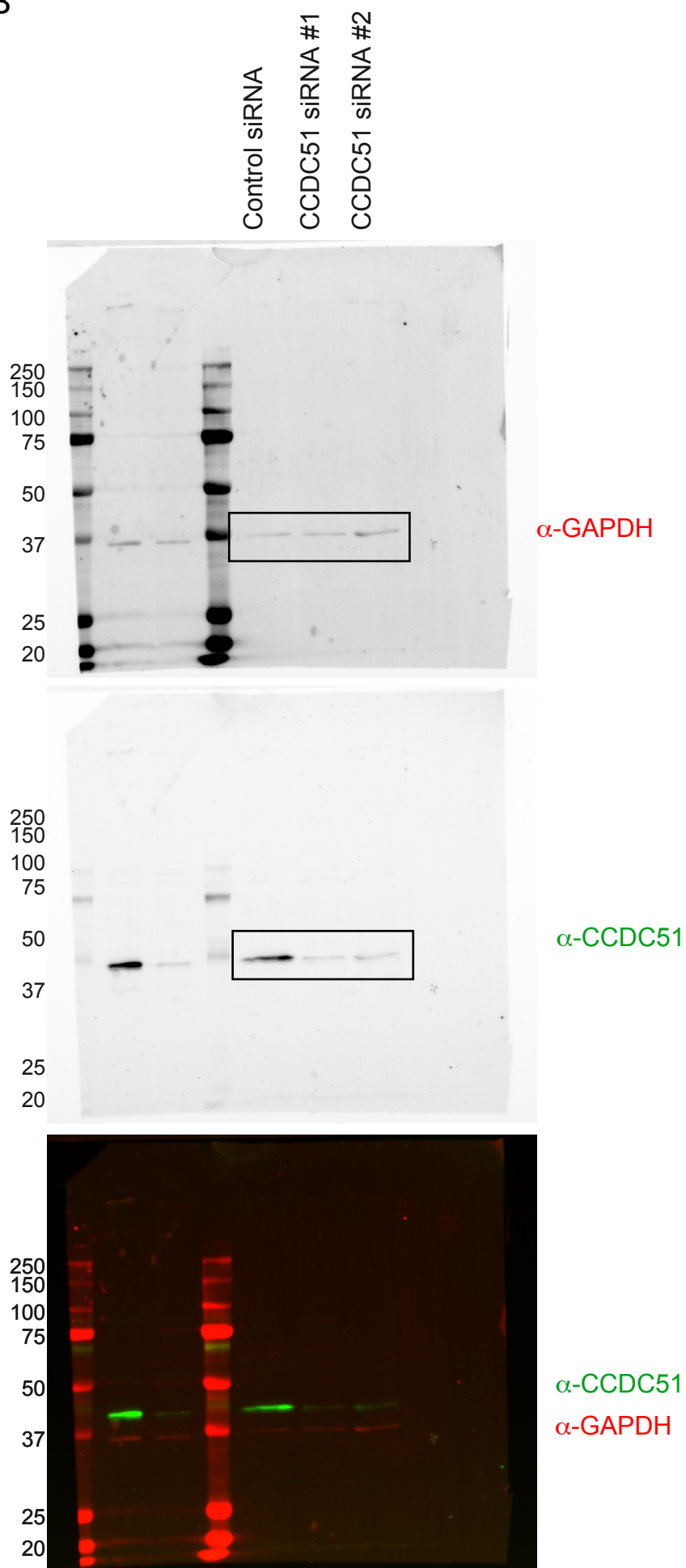

Supplement: SourceData F2 — is the source file for Fig. 2. [file jcb_202403140_sourcedataf2.pdf]

Source Data FS2

Fig. S2B

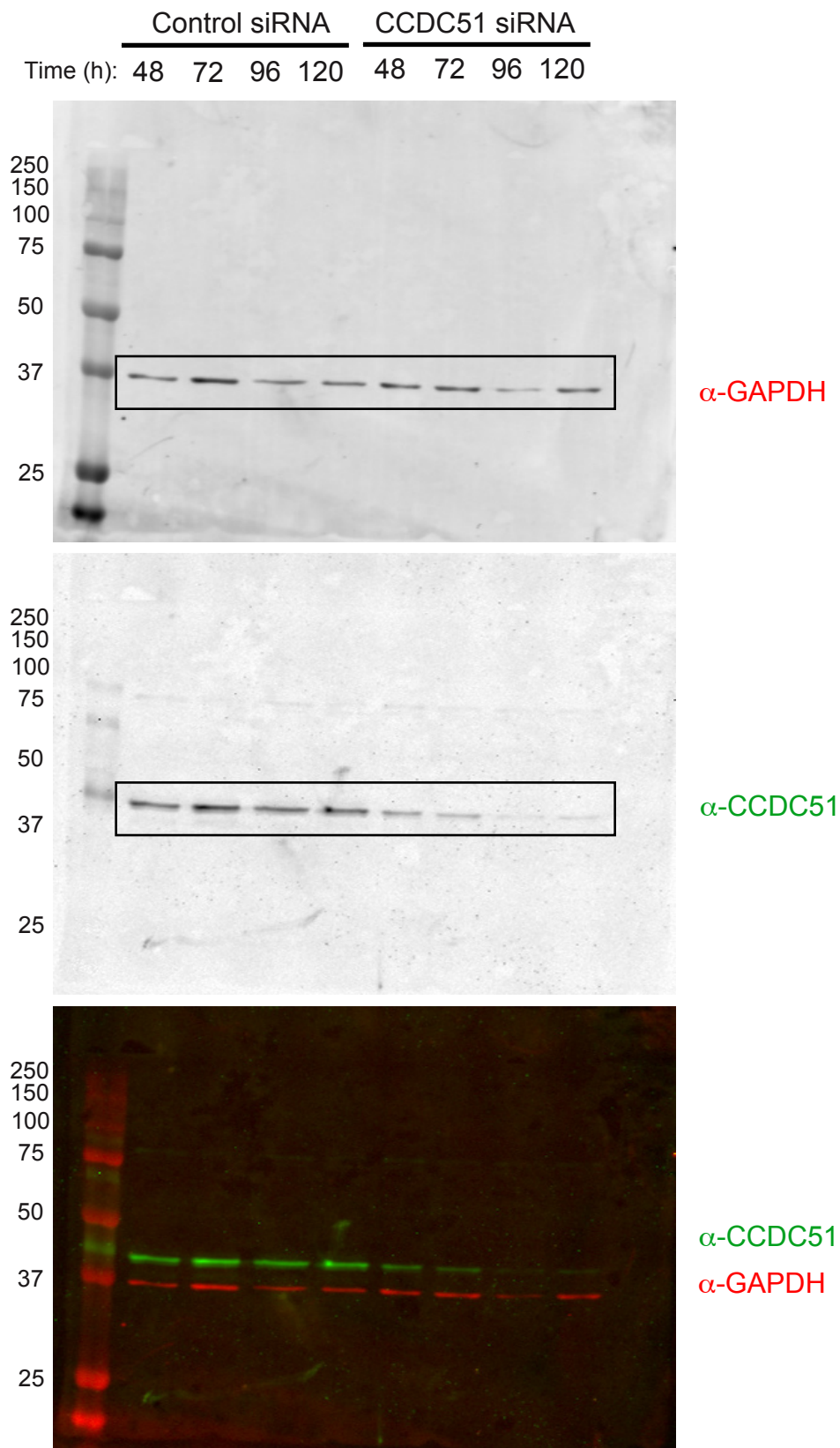

Fig. S2C

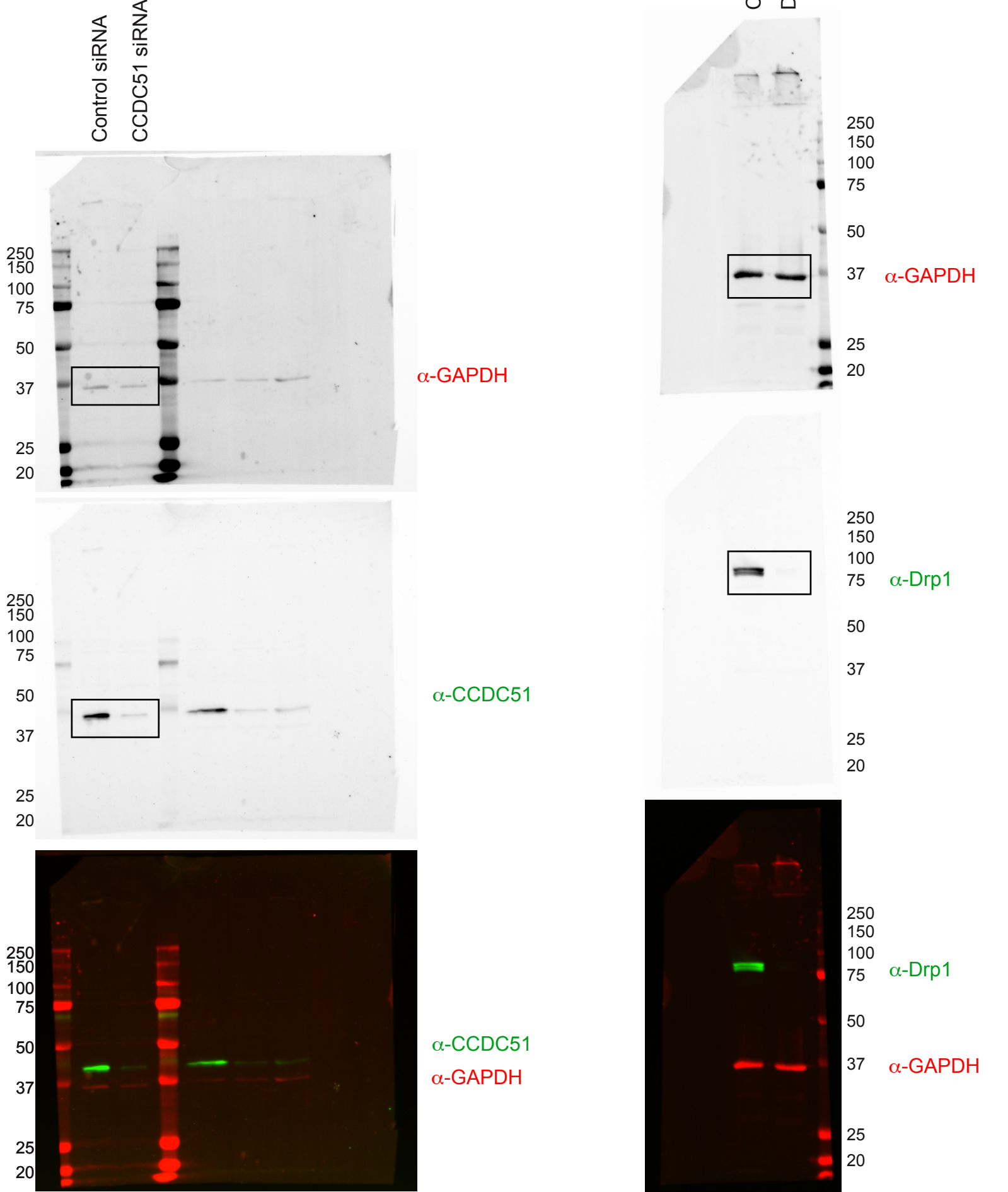

Source Data FS2 (cont.)

Fig. S2E

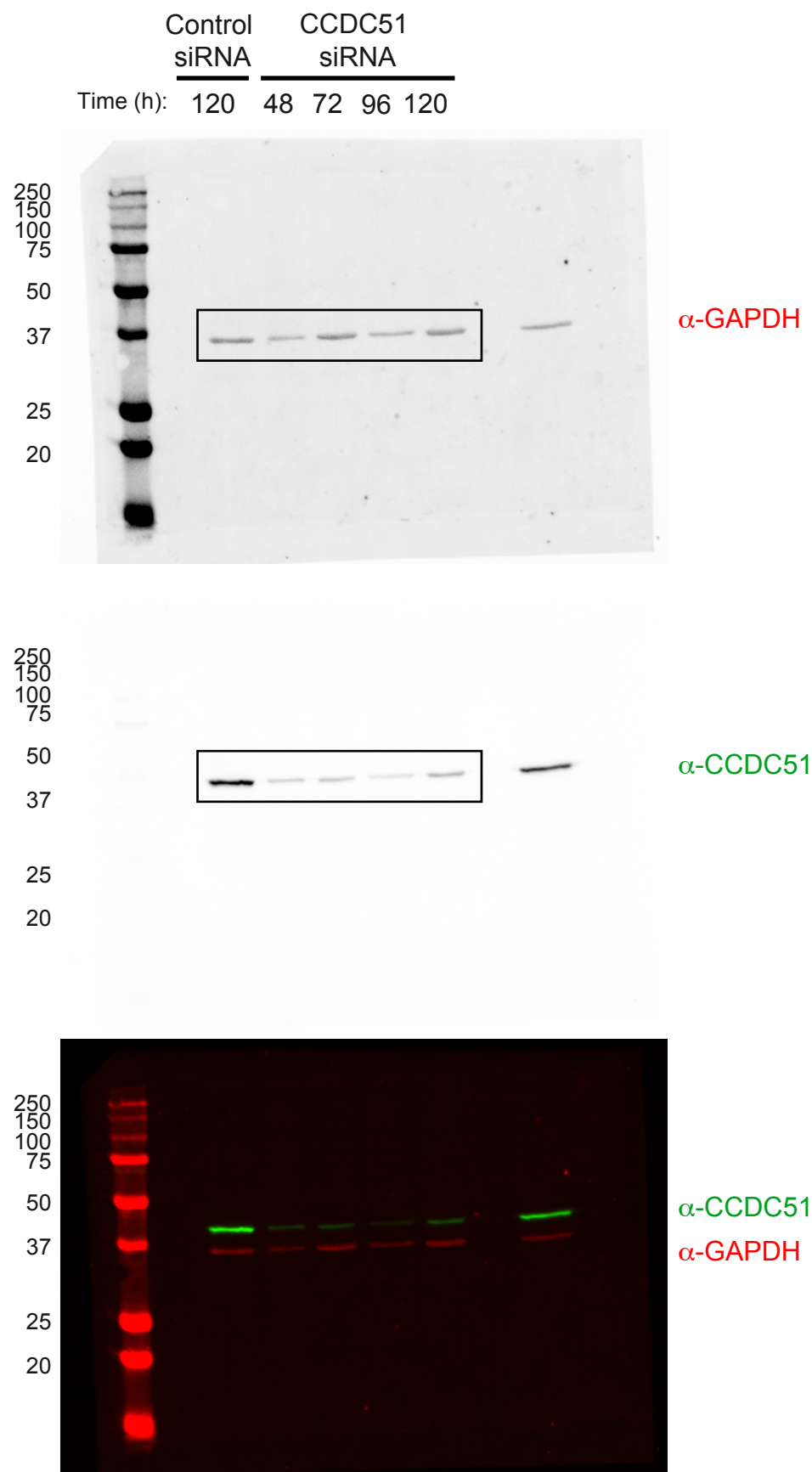

Supplement: SourceData FS2 — is the source file for Fig. S2. [file jcb_202403140_sourcedatafs2.pdf]
